# Hypoglycemia Induces Diabetic Macrovascular Endothelial Dysfunction via Endothelial Cell PANoptosis, Macrophage Polarization, and VSMC Fibrosis

**DOI:** 10.1002/advs.202414530

**Published:** 2025-07-12

**Authors:** Deyu Zuo, Yuce Peng, Guozhi Zhao, Zhesheng Cheng, Minghao Luo, Dingyi Lv, Shuting Chang, Na Li, Yanyao Huang, Xunjia Li, An He

**Affiliations:** ^1^ Division of Cardiology The First Affiliated Hospital of Chongqing Medical University Chongqing 400016 China; ^2^ Cardiovascular Disease Laboratory of Chongqing Medical University Chongqing 400016 China; ^3^ Department of Rehabilitation Medicine The First Affiliated Hospital of Chongqing University of Chinese Medicine Chongqing Traditional Chinese Medicine Hospital Chongqing 400021 China; ^4^ Department of Nephrology The First Affiliated Hospital of Chongqing University of Chinese Medicine Chongqing Traditional Chinese Medicine Hospital Chongqing 400021 China; ^5^ Department of Research and Development Chongqing Precision Medical Industry Technology Research Institute Chongqing 400000 China; ^6^ Departments of Urology surgery The First Affiliated Hospital of Chongqing Medical University Chongqing 400000 China; ^7^ Department of Pharmaceutical Sciences College of Pharmacy and Health Sciences St. John's University Queens NY 11439 USA

**Keywords:** ANGPTL4, fibrosis, hypoglycemia, PANoptosis, proinflammatory polarization

## Abstract

Hypoglycemia is a commonly neglected complication in elderly diabetic patients, which can lead to cardiovascular events. Endothelial cell dysfunction is the primary inducer of cardiovascular events, and it is associated with hypoglycemia‐triggered cytokine release and inflammatory programmed cell death. A comprehensive understanding of lineage‐specific variations in pathological vascular changes is essential to mitigate cardiovascular events and ensure therapeutic efficacy. Herein, unbiased clustering analyses and single‐nucleus RNA sequencing are performed on cells of the thoracic aorta in *db/db* and insulin‐induced hypoglycemic *db/db* mice. Comparative analyses show changes in lineage‐specific genes, subpopulation composition, intercellular communication, and molecular biology in hypoglycemic diabetic mice. The analyses also revealed the changes of different cells, particularly endothelial cell PANoptosis, macrophage inflammatory polarization, and vascular smooth muscle cell (VSMC) fibrosis. Pseudo‐time sequencing, differential expression, and regulation network analyses revealed the association of potential hub genes *Klf2*, *ETS2*, *Elavl1*, *C3*, and *Nr4a1* with the mentioned pathological processes. It is demonstrated that hypoglycemia induces VSMC fibrosis in vivo, whereas Angptl4 knockdown can attenuate VSMC fibrosis in vitro. These findings demonstrate the hypoglycemic macroangiopathy mechanism and provide important references for future disease intervention and treatment.

## Graphical abstract

This study investigates hypoglycemia‐induced diabetic macrovascular endothelial dysfunction. It reveals that hypoglycemia triggers ZBP1‐dependent PANoptosis of endothelial cells, proinflammatory polarization of macrophages, and fibrosis of vascular smooth muscle cells (VSMCs) in diabetic mice. Key regulators are identified, with ANGPTL4 validated as a pro‐fibrotic activator. The findings elucidate cellular mechanisms and offer therapeutic insights for hypoglycemia‐related cardiovascular events.

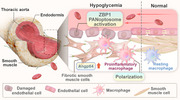

## 1 Introduction

With the global increase of the aging population, there has been a surge in the number of elderly diabetic patients, with 127 million people worldwide.^[^ ^1^, ^2^ ^]^ Elderly patients with diabetes are more likely to develop hypoglycemia than younger patients, which can lead to short‐ and long‐term adverse events and increased mortality.^[^ ^3^, ^4^ ^]^ The International Hypoglycaemia Study Group (IHSG) level 2 (glucose < 3.0 mmol L^−1^) and level 3 (severe) are associated with increased risks of cardiovascular events in type 2 diabetes mellitus (T2DM), such as myocardial ischemia and infarction, heart failure, and arrhythmias.^[^ ^4^ ^]^ Although hypoglycemia‐induced cardiovascular events have been intensively studied,^[^ ^5^, ^6^ ^]^ the underlying mechanisms are unclear.

Hypoglycemia increases circulating inflammatory mediators and atherogenic markers, which are related to endothelial cell dysfunction, manifested as a disorder of endothelium‐dependent vasodilation and cardiovascular diseases in diabetic patients.^[^ ^7^, ^8^, ^9^ ^]^ A previous study revealed that proinflammatory programmed cell death (PCD) caused by hypoglycemia is one of the important reasons for endothelial dysfunction and the source of cytokine release.^[^ ^10^ ^]^ The pattern of proinflammatory PCD includes pyroptosis, necroptosis, and PANoptosis.^[^ ^11^ ^]^ Pyroptosis has been reported in hypoglycemia‐induced endothelial dysfunction.^[^ ^10^ ^]^ An in‐depth elucidation of the role of other types of proinflammatory PCD in endothelial dysfunction in diabetic patients associated with hypoglycemia is fundamental for developing novel therapies to effectively avoid or alleviate hypoglycemia‐induced cardiovascular diseases.

Besides endothelial cells (ECs), macrophages and vascular smooth muscle cells (VSMCs) are also associated with hypoglycemia‐induced cardiovascular events. Macrophages are local immune cells that exist across organs and perform various crucial tasks for tissue homeostasis.^[^ ^12^ ^]^ They facilitate vascular inflammation, which is the main factor of global mortality and morbidity.^[^ ^13^, ^14^ ^]^ However, vascular macrophage polarization in the process of hypoglycemia is poorly understood. VSMCs are closely associated with endothelial dysfunction in vascular inflammation,^[^ ^15^ ^]^ which is detected in hypoglycemia through increased levels of proinflammatory cytokines in blood circulation, particularly interleukin (IL)‐6.^[^ ^16^ ^]^ IL‐6 produced by VSMCs within vessel walls is highly correlated with cardiovascular risks.^[^ ^17^ ^]^ The role of VSMCs in diabetes with hypoglycemia remains to be explored.

Previous studies have applied molecular biology to examine pathophysiological alterations in vascular function during hypoglycemia at the tissue level.^[^ ^8^, ^10^, ^16^ ^]^ However, cell type‐specific variations could not be detected from bulk data. This limitation can be solved by single‐cell RNA sequencing (scRNA‐seq) or single‐nucleus RNA‐seq (snRNA‐seq), which allow for unbiased dissection of cellular alterations at remarkable resolution. snRNA‐seq has been effectively applied to compare diabetic and nondiabetic ECs in adults.^[^ ^18^ ^]^ However, a lack of research exists on the pathophysiology of macrophages and VSMCs under hypoglycemia in the thoracic aorta of diabetic animal models. Integrated analysis of snRNA‐seq combined with molecular biology data could improve knowledge of cellular and molecular changes of hypoglycemia.

This study aimed to assess the impact of hypoglycemia on the cardiovascular system, focusing on ECs, macrophages, and VSMCs of the thoracic aorta in *db/db* and hypoglycemia *db/db* mice. Single‐nucleus RNA‐seq and comparative analyses were applied to delineate lineage‐specific variations in gene expression, subpopulation composition, and intercellular communication in thoracic aorta tissues. This study prioritized potential key genes during the transition toward the proinflammatory PCD status of ECs, macrophage proinflammatory polarization, and VSMC fibrosis, which were validated with functional and molecular biology experiments. Activation of human VSMC fibrosis could be suppressed by an in vitro silencing of ANGPTL4, suggesting that ANGPTL4 may function as a transcription repressor in vascular fibrosis. This research is expected to help mitigate hypoglycemia progression in aging diabetic patients and lessening hypoglycemia‐induced cardiovascular events.

## 2 Results

### 2.1 scRNA‐Seq on Thoracic Aorta Tissues from *db/db* (DM) and Insulin‐Induced Hypoglycemic (HDM) *db/db* Mice

**Figure 1:**
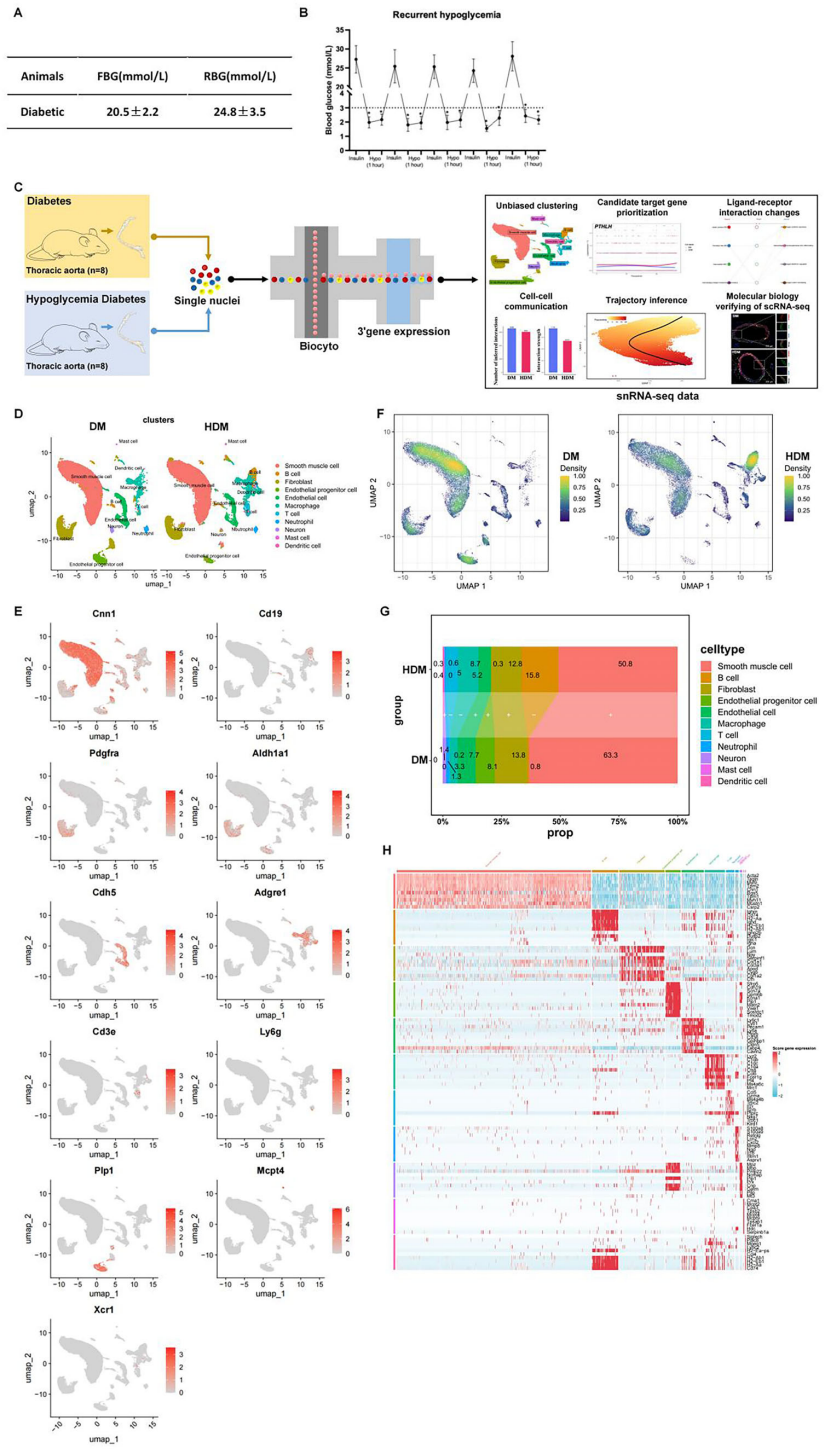
Variations in the relative proportion of each cell type of thoracic aorta tissues in DM and HDM groups. A,B) Construction of a *db/db* mouse hypoglycemic model. C) Scheme of the study design. Thoracic aorta tissues of mice in DM and HDM groups were collected for snRNA‐seq detection and analysis (n = 8). D) Unbiased clustering of nuclei from DM and HDM samples identified 11 major cell types. E) UMAP shows the expression of established marker genes in each cell type in the DM and HDM groups. F) Comparison of the nucleus density in the UMAP space between the two conditions reveals remarkable changes in the relative proportion of cell types in HDM. G) Changes in the relative proportion of each cell type in DM and HDM groups. +, expansion; –, contraction. H) Heatmap showing the molecular signature of each lineage.

Before investigating the hypoglycemia effects on ECs, macrophages, and VSMCs, a diabetic mouse hypoglycemic model was established following the procedures of previous studies^[^ ^10^, ^19^ ^]^ (**Figure** **1**A,B). Thoracic aorta tissues of DM and HDM mice were collected for snRNA‐seq (n = 8). Newly digested cells were collected from thoracic aorta tissues to prepare single‐cell suspensions using Biocyto for scRNA‐seq assay (Figure 1C). Cells were clustered, and results were visualized using unsupervised clustering and Unified Manifold Approximation and Projection (UMAP). Eleven subpopulations were obtained, from which 11 major cell types were identified based on the expression of classical markers (Figure 1D–F),^[^ ^20^ ^]^ with each cluster derived from six samples. Based on the expression of classical markers, 11 clusters were defined as SMCs (marked with *Cnn1*), B cells (marked with *Cd19*), fibroblasts (marked with *Pdgfra*), endothelial progenitor cells (EPC; marked with *Aldh1a1*), EC (marked with *Cdh5*), macrophages (marked with *Adgre1*), T cells (marked with *Cd3e*), neutrophils (marked with *Ly6g*), neurons (marked with *Plp1*), mast cells (marked with *Mcpt4*), and dendritic cells (DC; marked with *Xcr1*). After hypoglycemia induction, the proportion of vascular‐related cell lineages, including ECs and SMCs, significantly decreased, whereas the proportions of macrophage and fibroblast significantly increased in the inflammatory cell lineages (Figure 1G). The molecular characteristics of each cell lineage are shown in heatmaps (Figure 1H).

### 2.2 ECs

#### 2.2.1 EC‐Specific Regulatory Changes in the Thoracic Aorta of HDM

**Figure 2:**
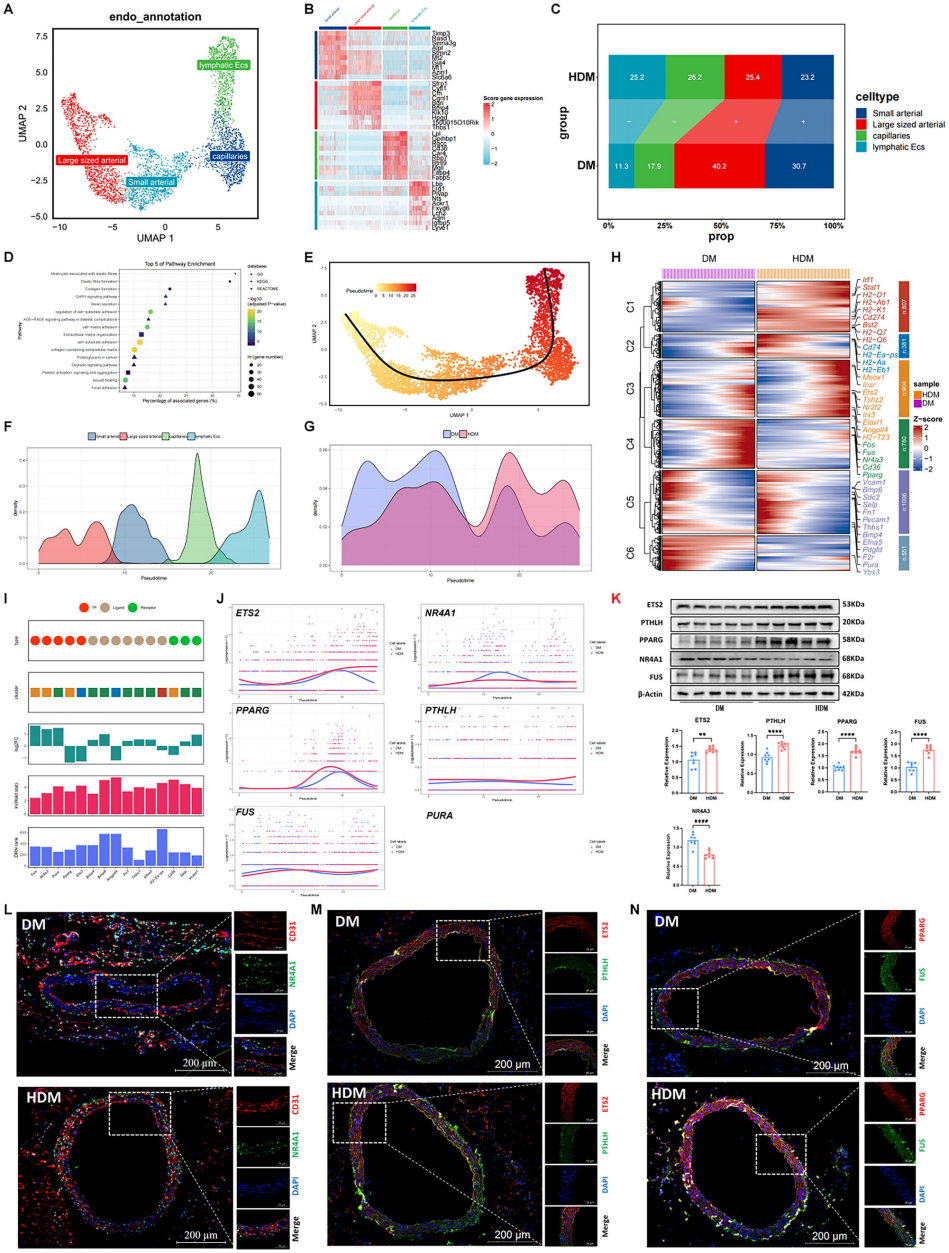
Endothelial cell‐specific regulatory changes in the inflammatory condition of HDM. A) UMAP plot presenting the endothelial cell (EC) subpopulation. B) Heatmap screening of the molecular signatures of each EC subpopulation. C) Relative proportion of each subpopulation in ECs from each condition. +, expansion; –, contraction. D) Representative terms enriched in significantly upregulated genes in ECs from HDM compared to DM. *P* < 0.05, hypergeometric test. E) Slingshot is used to reconstruct cell trajectories in transition to inflammatory ECs. F) Density curve showing the distribution of four EC subpopulations. G) Distribution density curves of EC subpopulations in DM and HDM. H) Heatmaps screening the expression dynamics of 4429 genes with significantly different patterns along the trajectory between the two conditions. These genes were detected by differential expression pattern analysis using the “conditionTest” function of tradeSeq and were categorized into six gene clusters by hierarchical clustering. Significance was set at *P* < 0.05. I) Prioritized key genes based on the results of three independent analyses, including differences in expression patterns, FC of expression levels, and centrality change in GRNs. DRN rank: gene ranking based on the centrality change in GRNs obtained by differential GRN analysis. log_2_FC: log_2_FC of expression levels in ECs. Wald stat: natural logarithm of the statistics of differential expression pattern analysis. Only genes encoding TFs, ligands, and receptors were considered. J) Smoothed expression curves of representative candidate genes along the trajectory under both conditions. K) Western blot analysis of protein levels of ETS2, PPARG, PLTLH, NR4A1, and FUS in the DM group (n = 8) and HDM group (n = 8). ^**^ *P* < 0.01, Wilcoxon rank‐sum test. L–N) Immunofluorescence staining showing the presence of the above genes in aortic tissues from DM and HDM mice. Scale bar, 200 µm.

Unbiased clustering divides ECs into four subpopulations: small arterial, large‐sized arterial, capillaries, and lymphatic ECs (**Figure** **2**A). Large‐sized arterial ECs expressed high levels of genes associated with atherosclerosis inflammatory markers, such as *BMP4* (encoding bone morphogenetic 4; a marker of atherosclerosis) and *HPGD* (encoding 15‐hydroxyprostaglandin dehydrogenase; a marker of inflammation),^[^ ^21^, ^22^ ^]^ indicating a proinflammatory state (Figure 2B). Lymphatic ECs expressed high levels of proinflammatory genes, such as *LCN2* (encoding lipocalin‐2; an enhancer of inflammation) and *PLVAP* (encoding plasma membrane vesicle‐associated; a marker of vascular injury),^[^ ^23^, ^24^ ^]^ indicating a proinflammatory and injury state (Figure 2B). Consistent with this phenomenon, the relative proportions of lymphatic and large‐sized arterial ECs in HDM were enlarged and shrunk, respectively (Figure 2C). DEsingle was applied to detect differentially expressed genes (DEGs) based on snRNA‐seq data in HDM versus DM for each lineage.^[^ ^25^ ^]^ For ECs, 2592 genes were significantly upregulated, and 130 genes were significantly downregulated (|log_2_FC| >1; *P* < 0.05). Upregulated gene enrichment is involved in signaling pathways associated with PCD and endothelial dysfunction (e.g., AGE‐RAGE signaling pathway), vasoconstriction (e.g., renin secretion pathway), energy metabolism (e.g., GnRH signaling pathway), and fibrosis (e.g., elastic fiber formation; Figure 2D). These results in the gene level revealed that hypoglycemia can trigger inflammation and cell death in ECs in a diabetic rodent model.^[^ ^8^, ^9^, ^10^ ^]^

#### 2.2.2 Transcriptomic Dynamics During the Transition of ECs Toward an Inflammation State in HDM

To examine the dynamic transcriptome of HDM ECs during their transition to an inflammatory state, the trajectory of nuclei of ECs in the pseudo‐temporal ordering was reconstructed using Slingshot (Figure 2E).^[^ ^26^ ^]^ Inflammatory ECs in large‐sized arterial ECs appeared in the relatively anterior stage with the pseudo‐time trajectory (Figure 2F). There were significant differences in the pseudo‐temporal distribution between DM and HDM (Figure 2G; *P* < 2.2 e^−16^, Kolmogorov–Smirnov test). Using tradeSeq,^[^ ^27^ ^]^ genes that showed significantly different expression patterns along the trajectory in the two cases were identified and clustered into six gene clusters (Figure 2H; *P* < 0.05). Based on the differential expression model adjusted *P*‐value of <0.05,^[^ ^28^ ^]^ multiple changes in expression level between DM and HDM states (|log_2_FC| > 0.5) were adjusted. Three independent analyses were performed, including the difference in expression patterns along the trajectory (*P* < 0.05), the fold change (FC) of expression levels between conditions (|log_2_FC| > 1), and the centrality change in gene regulatory networks (GRNs; DRN rank < 1000; Figure 2I).^[^ ^28^ ^]^ Only genes encoding transcription factors (TFs), ligands, and receptors were considered. The roles of most genes in EC transition toward inflammation, PCD, fibrosis, and reduced endothelial inflammation protection have been previously recognized as *ETS2* (encoding oncogene ets‐2),^[^ ^29^ ^]^ *NR4A1* (encoding nuclear receptor subfamily 4 group A member 1),^[^ ^30^ ^]^ *PURA* (encoding purine‐rich element binding protein alpha), and *PPARG* (encoding peroxisome proliferator‐activated receptor γ).^[^ ^31^, ^32^ ^]^ The gene that symbolizes neurodegenerative diseases, such as *FUS* (encoding fused in sarcoma),^[^ ^33^ ^]^ and signifies electrolyte disturbance, such as *PTHLH* (encoding parathyroid hormone‐related protein 1), has been discovered (Figure 2J).^[^ ^34^ ^]^ Immunoblotting confirmed that the protein levels of ETS2, PLTLH, PPARG, and FUS significantly increased and that NR4A1 and PURA significantly decreased in thoracic aorta tissues from HDM (Figure 2K). Immunofluorescence staining not only confirmed the presence of the above gene expression in DM and HDM tissue sections but also demonstrated their location in ECs (as confirmed by CD31; Figure 2L). These data showed that hypoglycemia, on the one hand, encourages inflammation and, on the other hand, weakens the anti‐inflammatory protein in diabetic ECs.

#### 2.2.3 Pyroptosis, Apoptosis, and Necroptosis (PANoptosis) of Diabetic ECs are Ignited by Hypoglycemia

**Figure 3:**
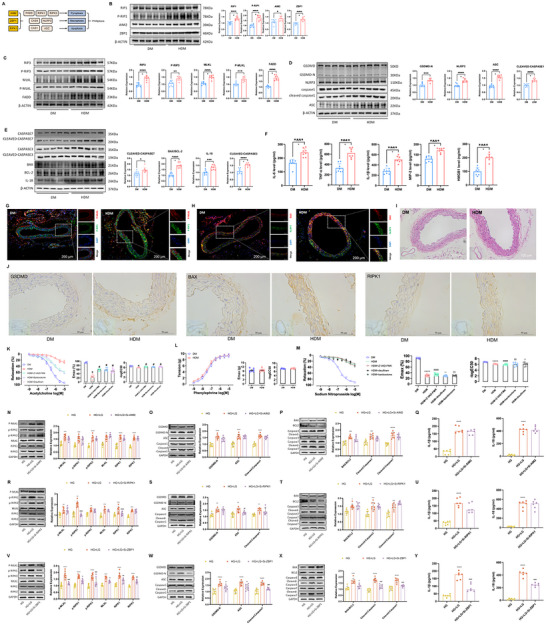
Hypoglycemia‐induced endothelial dysfunction by triggering EC PANoptosis. A) Scheme of PANoptosome (AIM2, ZBP1, and RIPK1)‐induced PANoptosis. B) Western blot assays confirm that protein levels of PANoptosis sensors AIM2, ZBP1, and RIPK1 change significantly in thoracic aorta tissues from HDM (n = 8) compared to DM (n = 8). ^*^ *P* < 0.05; ^**^ *P* < 0.01, Wilcoxon rank‐sum test. C–E) Western blot assays confirming the significant change of PANoptosis protein levels in thoracic aorta tissues from HDM (n = 8) compared to DM (n = 8). ^*^ *P* < 0.05; ^**^ *P* < 0.01, Wilcoxon rank‐sum test. F) ELISA confirming the significant change of cytokine levels of proinflammatory PCD in thoracic aorta tissues from HDM (n = 8) compared to DM (n = 8). ^*^ *P* < 0.05; ^**^ *P* < 0.01, Wilcoxon rank‐sum test. G,H) Immunofluorescence staining showing the presence of PANoptosis proteins in thoracic aorta tissues from DM and HDM mice. Scale bar, 200 µm. I) Representative micrographs of immunohistochemical staining for GSDMD, BAX, and RIPK1 in HDM and DM thoracic aortic tissue. Scale bar, 50 µm. J) H&E staining confirms the continuity of the vascular endothelium in thoracic aorta tissues from DM and HDM. Scale bar, 100 µm. K) Left, Endothelium‐dependent vasodilation in DM, Z‐VAD‐FMK + HDM, bardoxolone + HDM, disulfiram + HDM, and HDM thoracic aorta rings (n = 8). PANoptosis inhibitors have a similar effect to attenuate the curve of endothelium‐dependent vasodilation. Right, EC_50_ of thoracic aortic rings in the above group. PANoptosis inhibitors similarly attenuate the decreasing EC_50_ and maximum diastolic rate of the thoracic aorta under hypoglycemia. ^*^ *P* < 0.05, HDM versus DM, ^#^Z‐VAD‐FMK + HDM, bardoxolone + HDM, disulfiram + HDM versus HDM; *P* < 0.05, Wilcoxon rank‐sum test. L) Left, Vasoconstriction in DM and HDM thoracic aorta aortic rings (n = 8). Hypoglycemia did not significantly change the vasoconstriction of the thoracic aorta. Right, EC_50_ of thoracic aortic rings in DM and HDM. Hypoglycemia did not significantly change the EC_50_ and maximum constrictive rate of the thoracic aorta in HDM compared to DM. M) Left, Vascular smooth muscle vasodilation in DM, HDM, HDM + Z‐VAD‐FMK, HDM + bardoxolone, and HDM + disulfiram endothelium‐free thoracic aorta rings (n = 8). Z‐VAD‐FMK, bardoxolone or disulfiram has an effect to attenuate the curve of vascular smooth muscle vasodilation. Right, EC_50 _of endothelium‐free thoracic aortic rings in the above group. Z‐VAD‐FMK, bardoxolone or disulfiram has no effect to attenuate the EC_50_ and maximum diastolic rate of the thoracic aorta under hypoglycemia. ^****^ *P* < 0.0001, HDM versus DM, ^####^HDM + Z‐VAD‐FMK versus DM; *P* < 0.0001, ^$$^HDM + bardoxolone versus DM; *P* < 0.0001, ^^HDM + disulfiram versus DM; *P* < 0.0001, Wilcoxon rank‐sum test. N–P,R–T,V–X) Western blot assays confirming the significant change of PANoptosis protein levels in MAEC, from low glucose treatment (n = 8) compared to high glucose cultured (n = 8). ^*^ *P* < 0.05; ^**^ *P* < 0.01; ^***^ *P* < 0.001; ^****^ *P* < 0.0001, si‐RIPK1 and si‐ZBP1 from low glucose treatment (n = 8) compared to low glucose treatment (n = 8). ^#^ *P* < 0.05; ^##^ *P* < 0.01; ^###^ *P* < 0.001; ^####^ *P* < 0.0001, Wilcoxon rank‐sum test. Q,U,Y) ELISA confirming the significant change of cytokine levels of PANoptosis in MAEC, from low glucose treatment (n = 8) compared to high glucose cultured (n = 8). ^****^ *P* < 0.0001, si‐ZBP1 from low glucose treatment (n = 8) compared to low glucose treatment (n = 8). ^####^ *P* < 0.0001, Wilcoxon rank‐sum test.

Hypoglycemia has sustained proinflammatory and deteriorative effects on endothelium‐dependent vasodilation of peripheral arteries in T2DM.^[^ ^8^ ^]^ To further interpret the pattern during the transition toward an inflammatory PCD state of EC in HDM, immunoblotting was applied to determine the protein level of PCD. PANoptosis is a newly recognized inflammatory PCD pattern,^[^ ^35^ ^]^ driven through a multiprotein complex and activated by sensors AIM2, ZBP1, and RIPK1 to form a flexible skeleton in recruiting PANoptosis‐related proteins (**Figure** **3**A). The immunoblotting results indicated activation of PANoptosis in the thoracic aorta of HDM manifested as increased levels of AIM2, ZBP1, and RIPK1 (sensors and essential members of PANoptosome), active forms of caspase‐1 and GSDMD (pyroptosis markers), active forms of caspase‐3, BAX, and BCL‐2 (apoptosis markers), and phosphorylation of MLKL, RIPK1, and RIPK3 (necroptosis markers; Figure 3B–D). Enzyme‐linked immunosorbent assay (ELISA) was applied to determine the significant increase of cytokines, such as IL‐18, IL‐6, IL‐1β, tumor necrosis factor‐α, macrophage inflammatory protein‐2, and high mobility group box‐1, produced from proinflammatory PCD of diabetic thoracic aorta tissues under hypoglycemia (Figure 3F). Immunofluorescence and immunohistochemistry revealed that hypoglycemia aggravated PANoptosis protein expression in the vascular endothelium (Figure 3G,H,J). Results of hematoxylin‐and‐eosin (H&E) staining of diabetic thoracic aorta tissues showed that the continuity of the vascular endothelium in HDM significantly decreased compared to DM (Figure 3I). The above results suggest that hypoglycemia induces endothelial cell dysfunction by evoking PANoptosis.

To comprehensively evaluate the endothelial cell dysfunction caused by hypoglycemia‐induced PANoptosis, endothelial‐dependent vasodilation was analyzed using Multi Wire Myograph. Relaxant responses to acetylcholine (Ach) in aortic segments of insulin‐treated HDM mice were significantly higher than those in the DM group, whereas hypoglycemia in these vessels increased the median effective dose (EC_50_) and maximum diastolic rate. Separate usage of the apoptosis inhibitor Z‐VAD‐FMK, the necroptosis inhibitor bardoxolone, or pyroptosis inhibitor disulfiram exhibits improvement in endothelial cell dysfunction caused by hypoglycemia (Figure 3I). Furthermore, vasoconstrictive responses to phenyl epinephrine (PE) in aortic segments of insulin‐treated HDM mice lead to no significant change in EC_50_ and maximum constrictive rate (Figure 3J). To comprehensively evaluate the endothelial cell dysfunction caused by hypoglycemia‐induced PANoptosis, endothelia‐dependent vasodilation was analyzed using Multi Wire Myograph. Relaxant responses to acetylcholine (Ach) in aortic segments of insulin‐treated HDM mice were significantly higher than those in the DM group, whereas hypoglycemia in these vessels increased the median effective dose (EC_50_) and maximum diastolic rate. Separate usage of the apoptosis inhibitor Z‐VAD‐FMK, the necroptosis inhibitor bardoxolone, or the pyroptosis inhibitor disulfiram exhibits improvement in endothelial cell dysfunction caused by hypoglycemia (Figure 3K). Furthermore, vasoconstrictive responses to phenyl epinephrine (PE) in aortic segments of insulin‐treated HDM mice lead to no significant change in EC_50_ and maximum constrictive rate (Figure 3L). We also applied endothelial‐free thoracic aortic rings in vivo to confirm the effect of Z‐VAD‐FMK, disulfiram, and bardoxolone on smooth muscle cells of diabetic mice during hypoglycemia. In the endothelium‐free thoracic aorta rings, relaxant responses to sodium nitroprusside (SNP) in aortic segments of insulin‐treated HDM mice were significantly higher than those in the DM group, whereas hypoglycemia in these vessels increased the median effective dose (EC_50_) and maximum diastolic rate. Separate usage of the apoptosis inhibitor Z‐VAD‐FMK, necroptosis inhibitor bardoxolone, or pyroptosis inhibitor disulfiram has no effect to alleviate the smooth muscle cell dysfunction caused by hypoglycemia (Figure 3M). The above results suggest that hypoglycemia induces endothelial cell dysfunction by evoking PANoptosis. To further identify which specific sensor mediates PANoptosis in diabetic thoracic aorta tissues under hypoglycemia, small interfering (si)‐RNA and immunoblotting were applied to determine the function of PANoptosis sensors. The immunoblotting results indicated that only silencing of expression of ZBP1 in low glucose treatment endothelial cells can suppresses the expression of caspase‐1 and GSDMD (pyroptosis markers), caspase‐3, BAX, and BCL‐2 (apoptosis markers), and phosphorylation of MLKL, RIPK1, and RIPK3 (necroptosis markers) (Figure 3N–P,R–T,V–X). ELISA was applied to determine the significant increase of cytokines, like IL‐18 and IL‐1β, produced from PANoptosis of low glucose‐treated endothelial cells, and increased release of these cytokines can be suppressed by silencing ZBP1 expression (Figurs 3Q,U,Y,S1). These results suggested that hypoglycemia triggers PANoptosis of endothelial cells by mediating activation of ZBP1 PANoptosome.

### 2.3 Macrophages

#### 2.3.1 Relative Proportion of Lineage Subpopulations and Polarization Changes of Macrophages in HDM

**Figure 4:**
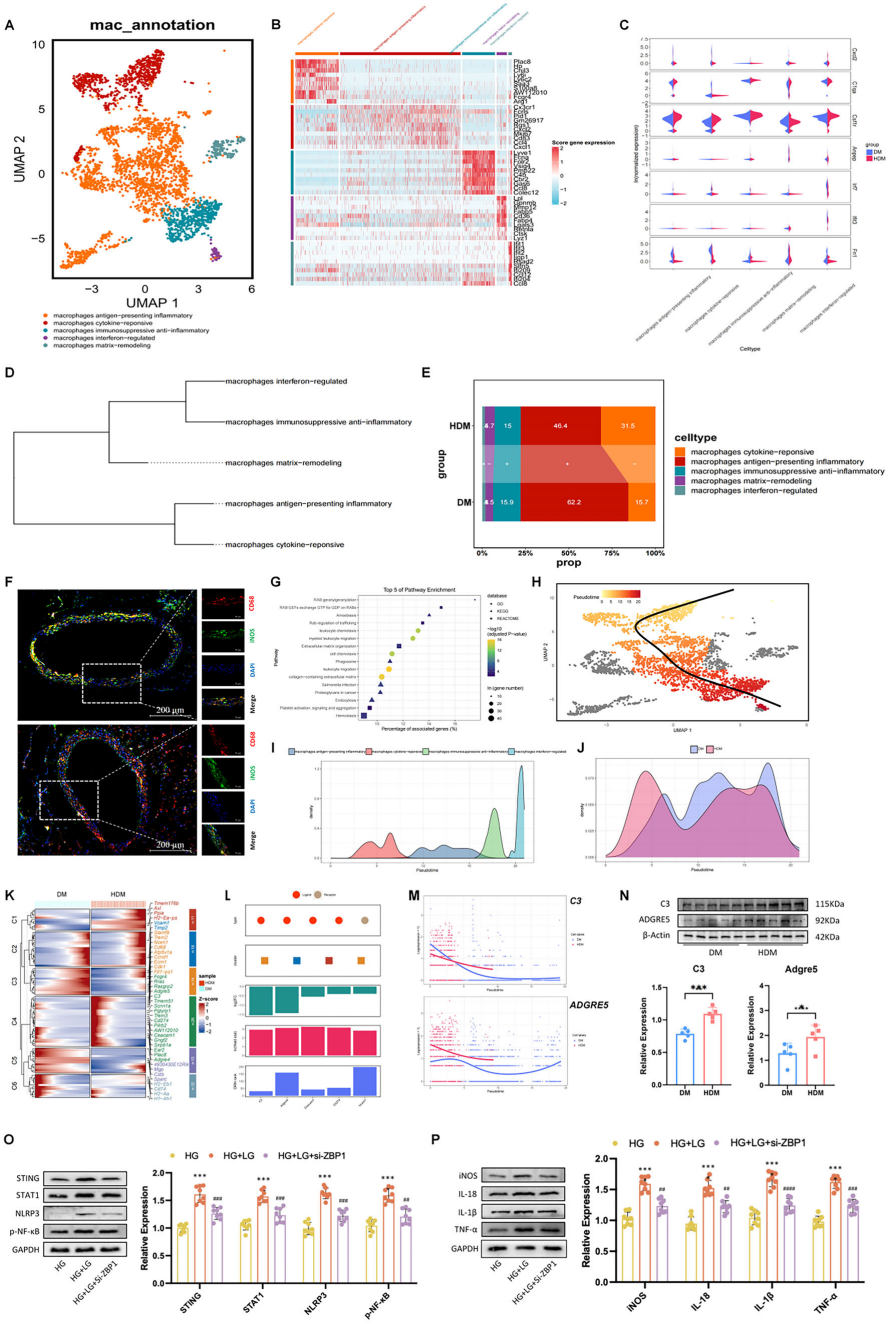
Macrophage‐specific regulatory changes in the inflammatory condition of HDM. A) UMAP plot showing the macrophage subpopulation. B) Heatmap presenting the molecular signatures of every macrophage subpopulation. C) Violin diagram of division showing the expression of activated macrophage markers. D) Hierarchical clustering of subpopulations. E) Relative proportion of each subpopulation in macrophages from each condition. +, expansion; –, contraction. F) Immunofluorescence staining showing the presence of the proinflammatory polarization of macrophages in thoracic aorta tissues from HDM and DM mice. Proinflammatory polarization and macrophages are labeled with iNOS and CD68, respectively. Scale bar, 200 µm. G) Representative terms enriched in upregulated genes in macrophages from HDM compared to DM. *P* < 0.05, hypergeometric test. H) Slingshot to reconstruct cell trajectories in transition to proinflammatory macrophages. I) Density curve showing the distribution of four subpopulations of macrophages. J) Distribution density curves of macrophage subpopulations in DM and HDM groups. K) Heatmap showing the 94 gene expression dynamics in DM and HDM, with their significantly different expression patterns. The conditionTest function of tradeSeq was used to analyze differential expression patterns, divided into six gene clusters using hierarchical clustering. The significance threshold was set at 0.05. L) Potential key genes prioritized based on the results of three independent analyses, including the difference in expression patterns, the FC of expression levels, and the centrality change in GRNs. M) Smoothed expression curves of representative candidate genes along the locus under both conditions. N) Western blot analysis of protein levels of C3 and Adrge5 in the DM group (n = 8) and HDM group (n = 8). ^**^ *P* < 0.01, Wilcoxon rank‐sum test. O,P) Western blot assays confirming the significant change of levels of signaling pathways and marker of proinflammatory polarization of macrophage in the co‐culture system from low glucose incubation (n = 8) compared to high glucose cultured (n = 8). ^***^ *P* < 0.001; ^****^ *P* < 0.0001, si‐ZBP1 + low glucose incubation versus low glucose incubation; ^##^ *P* < 0.01; ^###^ *P* < 0.001; ^####^ *P* < 0.0001, Wilcoxon rank‐sum test.

Based on biological function, unbiased clustering classifies macrophages into five subpopulations: antigen‐presenting inflammatory, cytokine‐responsive, immunosuppressive anti‐inflammatory, interferon (IFN)‐regulated, and matrix‐remodeling macrophages (**Figure** **4**A). Cytokine‐responsive macrophages expressed high levels of inflammatory marker genes such as *CHIL3* (encoding chitinase 3‐like protein 1; a marker of inflammatory state) and *SAA3* (encoding serum amyloid A3; a marker of acute phase of inflammatory stimulation), indicating that this macrophage subpopulation is at an inflammatory state.^[^ ^36^, ^37^ ^]^ Antigen‐presenting macrophages expressed high levels of immunosuppression genes, such as *RGS1* (encoding regulator of G protein signaling 1) and *CD83* (encoding cluster of differentiation 83), indicating that this subgroup was in an immunosuppressive state (Figure 4B).^[^ ^38^, ^39^ ^]^ Macrophages in HDM express the highest levels of immunoreaction markers, including CXC chemokine ligand 1, C1QA, IFN regulatory factor‐7, and FN1, representing the proinflammatory state of macrophages (Figure 4C).^[^ ^40^, ^41^, ^42^, ^43^ ^]^ Hierarchical clustering showed a close relationship between antigen‐presenting inflammatory, and cytokine‐responsive macrophages (Figure 4D). The increase in the relative proportion of cytokine‐responsive macrophages and the decrease in the relative proportion of antigen‐presenting macrophages in HDM might explain that hypoglycemia has proinflammatory effects in diabetes (Figure 4E). Macrophage polarization in the thoracic aorta of DM mice under hypoglycemia level was evaluated using immunofluorescence staining. The markers for macrophage proinflammatory polarization, such as inducible nitric oxide synthase (iNOS), significantly increased in thoracic aorta tissues of the HDM group (Figure 4F).^[^ ^44^ ^]^ In macrophages, DEsingle was also detected using DEGs between snRNA‐seq data in HDM and DM for each lineage.^[^ ^25^ ^]^ The expression of 190 genes was significantly upregulated, whereas 95 genes were significantly downregulated (|log_2_FC| > 1; *P* < 0.05). Consistent with the reported physiological process of immunoreaction, upregulated gene enrichment in pathogen‐associated molecular pattern invasion (e.g., amoebiasis and *Salmonella* infections), chemotaxis (e.g., leukocyte chemotaxis), migration (e.g., leukocyte migration), and endocytosis‐related signaling pathways (Figure 4G), these findings suggested that hypoglycemia might evoke inflammation in diabetes.^[^ ^8^, ^9^, ^10^ ^]^

#### 2.3.2 Macrophage Proinflammatory Polarization Regulatory Genes and their Changes in HDM

To explore the trajectory and evolution of macrophage differentiation during the transition to proinflammatory polarization in diabetic thoracic aorta under hypoglycemia, Slingshot was applied (Figure 4H).^[^ ^26^ ^]^ The sequence of cytokine‐responsive macrophages that express high levels of inflammatory marker genes occurred relatively early in the pseudo‐time locus (Figure 4I). There were significant differences in the pseudo‐temporal distribution between DM and HDM (Figure 4J; *P* < 2.2e^−16^, Kolmogorov‐Smirnov test). Using tradeSeq,^[^ ^27^ ^]^ genes that showed significantly different expression patterns along the tracks in the two cases were identified and clustered into six (Figure 4K; *P* < 0.05). The potential key genes were prioritized according to the above‐mentioned criteria in the part of ECs. Five priority genes were shown: *C3* (encoding complement component C3), *ADGRE5* (encoding adhesion G‐protein‐coupled receptor E5), *CEACAM1* (encoding carcinoembryonic antigen‐related cell adhesion molecule 1), *CD274* (encoding PCD 1 ligand 1), and *VCAM1* (encoding vascular cell adhesion molecule 1), all of them were identified for their roles in acute inflammation or immune responses (Figure 4L,M).^[^ ^45^, ^46^, ^47^, ^48^, ^49^ ^]^ Immunoblotting analysis confirmed that the protein levels of C3 and ADGRE5 significantly increased in HDM (Figure 4N). These findings implied that hypoglycemia could induce macrophage proinflammatory polarization in the diabetic thoracic aorta.

The signaling pathways related to proinflammatory polarization of macrophages have been well studied; however, the mechanism of hypoglycemia‐induced proinflammatory polarization in diabetes remains unknown. We investigated the changes in proinflammatory signaling pathways like stimulator of interferon genes (STING), signal transducer and activator of transcription 1 (STAT1), and nuclear factor kappa‐B (NF‐κβ)/NOD‐like receptor thermal protein domain‐associated protein 3 (NLRP3) during hypoglycemia.^[^ ^50^, ^51^, ^52^ ^]^ The immunoblotting results indicated proinflammatory polarization signaling pathway in the macrophages from the co‐culture system of endothelial cells and macrophages during low glucose incubation, manifested as increased levels of STING, STAT1, and NF‐κβ/NLRP3 (Figure 4O). In addition, increased protein expression of iNOS and cytokine release of IL‐18 and IL‐1β demonstrated the proinflammatory polarization of macrophages in the above co‐culture system (Figure 4P). Activated signaling pathways like STING and NF‐κβ/NLRP3 have been observed in hypoglycemia induced injured thoracic aorta of diabetic rodent model in our previous research.^[^ ^10^ ^]^ STAT1, the key regulating macrophages pro‐inflammatory polarization protein, was found to increase glucose metabolism in a recent study.^[^ ^50^ ^]^ These studies implied that the mechanism of hypoglycemia induced macrophage proinflammatory polarization might not rely exclusively on one specific signaling pathway. Therefore, identifying the regulation of macrophage pro‐inflammatory polarization under hypoglycemia is the fundamental approach to blocking its initiation and progression. It is well known that inflammatory programmed cell death of the endothelium evoked by hypoglycemia in diabetic rodent thoracic aorta releases a substantial amount of pro‐inflammatory cytokines and triggers oxidative stress.^[^ ^10^, ^19^ ^]^ And pro‐inflammatory cytokines and oxidative stress be considered as potent agonist for pro‐inflammatory macrophage polarization. Hence, we hypothesize that the PANoptosis of the endothelial cell triggered by hypoglycemia is closely related to macrophage pro‐inflammatory polarization. The immunoblotting results indicated that in the co‐culture system of si‐ZBP1 endothelial cells and macrophages, the increased expression of STING, STAT1, and NF‐κβ/NLRP3 was suppressed in macrophages after low glucose incubation (Figure 4O). Furthermore, the immunoblotting showed a decrease of macrophages, pro‐inflammatory polarization markers like inducible nitric oxide synthase (iNOS), TNF‐α, IL‐18, and IL‐1β (Figure 4P). Together, our results suggest that the inhibition of hypoglycemia induced PANoptosis in diabetic endothelial cells can attenuate macrophage pro‐inflammatory polarization.

### 2.4 VSMCs

#### 2.4.1 VSMC‐Specific Regulatory Changes in the Thoracic Aorta of HDM

**Figure 5:**
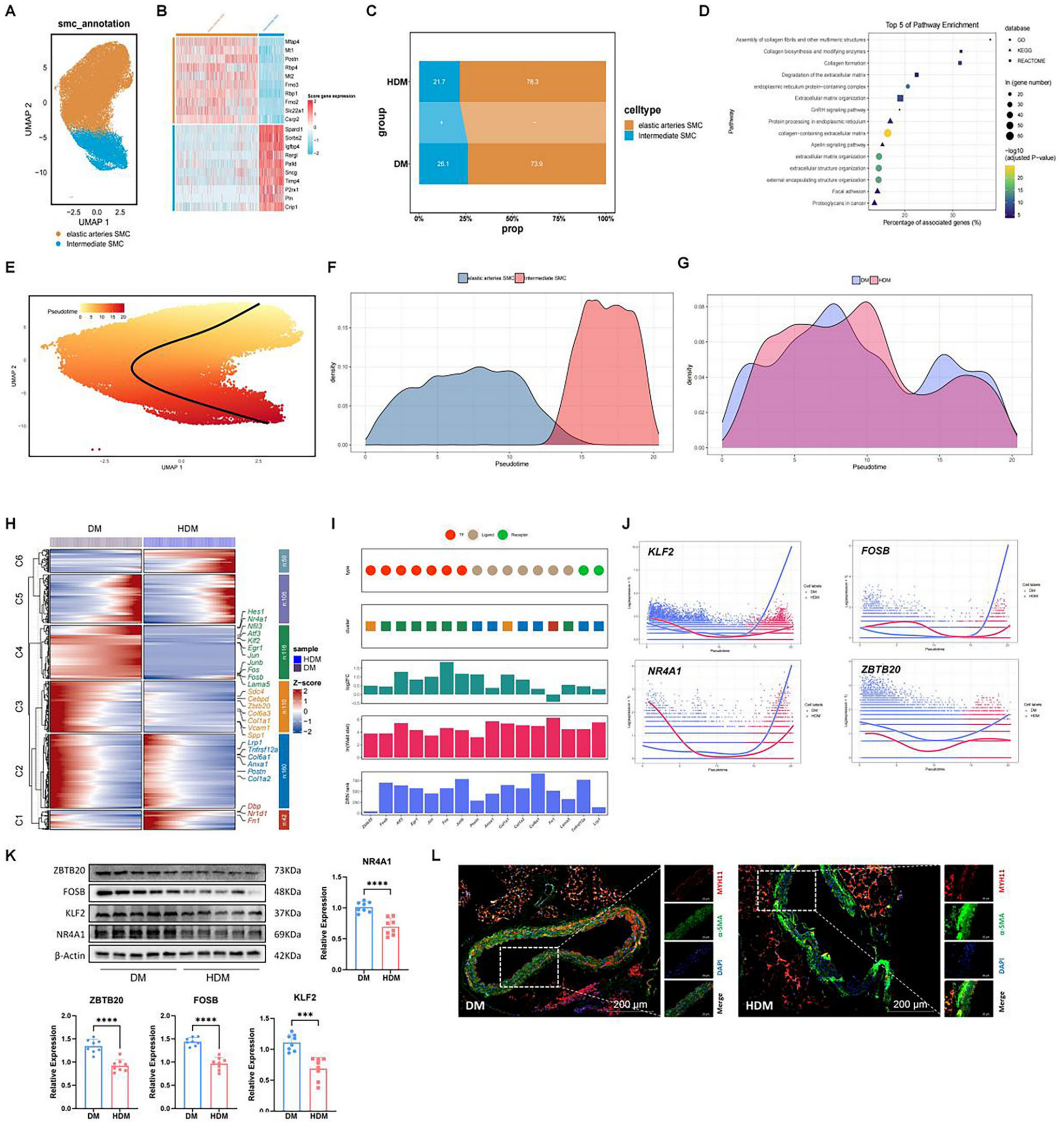
VSMC‐specific regulatory changes in the inflammatory condition of HDM. A) UMAP plot showing the VSMC subpopulation. B) Molecular signatures of each subpopulation of VSMC. C) Relative proportion of each subpopulation in VSMCs from each condition. +, expansion; –, contraction. D) Representative terms enriched in significantly upregulated genes in VSMCs from HDM compared to DM mice. *P* < 0.05, hypergeometric test. E) Slingshot to reconstruct cell trajectories in transition to inflammatory VSMCs. F) Distribution of two subpopulations of VSMCs. G) Distribution density curve of VSMC subpopulations in DM and HDM. H) Expression dynamics of 583 genes under two conditions, presenting significantly different expression patterns. These genes were analyzed for differential expression patterns using the “conditionTest” function of tradeSeq and divided into six gene clusters by hierarchical clustering. The significance threshold was set at 0.05. I) Potential key genes prioritized based on the results of three independent analyses, including the difference in expression patterns, the FC of expression levels, and the centrality change in GRNs. J) Expression curve along the trajectory of the representative candidate genes (*Klf2*, *Fosb*, *Nr4a1*, and *Zbtb20*) under both conditions. K) Western blot analysis of Klf2, Fosb, Nr4a1, and Zbtb20 protein levels in the DM group (n = 8) and HDM group (n = 8). ^**^ *P* < 0.01, Wilcoxon rank‐sum test. L) Immunofluorescence staining confirms SMC fibrosis in thoracic aorta tissues from HDM and DM. Fibrosis is labeled with α‐SMA and Myh11. Scale bar, 200 µm.

Unbiased clustering divides VSMCs into two subpopulations: elastic artery and intermediate SMCs (**Figure** **5**A). The first subpopulation expresses high levels of fibrotic marker genes such as *MFAP4* (encoding microfibril‐associated glycoprotein 4; a marker of atherosclerosis) and acute inflammatory response markers such as *MT1* (encoding metallothionin‐1; a marker of inflammation),^[^ ^54^, ^55^ ^]^ indicating that this subpopulation was moving toward fibrosis, the worst complication of inflammation.^[^ ^56^ ^]^ The expression of *SORBS2* (encoding sorbitol and SH3 domain 2 protein), which is negatively associated with congenital heart disease and atherosclerosis, indicates that the SMC subpopulation is in an inflammatory state (Figure 2B).^[^ ^57^ ^]^ Consistent with this phenomenon, the relative proportions of elastic artery SMCs increased, and those of intermediate SMCs reduced in HDM (Figure 5C). In SMCs, the expression of 155 genes was significantly upregulated, whereas 46 genes were significantly downregulated (|log_2_FC| >1; *P* < 0.05). Consistent with the reported pathophysiological changes in smooth muscle fibrosis and chronic inflammation, upregulated gene enrichment in signaling pathways is associated with fibrosis [e.g., extracellular matrix (ECM) remodeling], immune response (e.g., ECM), and energy metabolism (e.g., Apelin signaling pathway; Figure 5D). These bioinformatics analyses demonstrated that hypoglycemia might evoke inflammation in VSMCs in the thoracic aorta of a diabetic rodent model.^[^ ^8^, ^9^, ^10^ ^]^

#### 2.4.2 Transcriptomic Dynamics During the Transition of VSMC Toward an Inflammation State in HDM

To mine the dynamic transcriptome of HDM ECs during their transition to an inflammatory state, the trajectory through a pseudo‐temporal ordering of the nuclei of VSMCs was reconstructed using Slingshot (Figure 5E).^[^ ^26^ ^]^ Inflammatory VSMCs in elastic artery SMCs appeared in the relatively anterior stage along the pseudo‐time trajectory (Figure 5F). There were significant differences in the pseudo‐temporal distribution between DM and HDM (Figure 5G; *P* <2.2e^−16^, Kolmogorov‐Smirnov test). Using tradeSeq,^[^ ^27^ ^]^ genes that showed significantly different expression patterns along the trajectory in the two cases were identified and clustered into six (Figure 5H; *P* < 0.05). Based on the differential expression model *P* < 0.05,^[^ ^28^ ^]^ the multiple changes of the expression level between DM and HDM states (|log_2_FC| > 0.5) were adjusted. The potential key genes were prioritized according to the above‐mentioned criteria, similar to ECs (Figure 5I). The role of most genes in cardiovascular diseases has been reported, such as *KLF2* (encoding Krüppel‐like factor 2) and *NR4A1* (encoding nuclear receptor subfamily 4 group A1), which play a protective and inhibitory effect in atherosclerosis and inflammation.^[^ ^58^, ^59^ ^]^ *Zbtb20* (encoding zinc finger and BTB domain‐containing 20) has a protective effect in heart failure caused by myocardial hypertrophy,^[^ ^60^ ^]^ and *f osb* (encoding the fosB proto‐oncogene) for which the expression decreased, was associated with aneurysms^[^ ^61^ ^]^ (Figure 5J). Immunoblotting confirmed that the protein levels of Zbtb20 and FOSB significantly decreased in thoracic aorta tissues from HDM (Figure 5K). To further decipher the transcriptomic dynamics during the activation of aortic SMC inflammation in HDM, the marker of chronic inflammation and fibrosis through immunofluorescence was determined. α‐Smooth muscle actin (α‐SMA) increased in SMCs of HDM (Figure 5L). These results suggested that hypoglycemia aggravated SMC fibrosis in the thoracic aorta of diabetes mice by lowering the expression of protective genes and their encoding proteins.

#### 2.4.3 Changes in Intercellular Communication in HDM Thoracic Aorta Tissues Inferred from snRNA‐Seq Data

**Figure 6:**
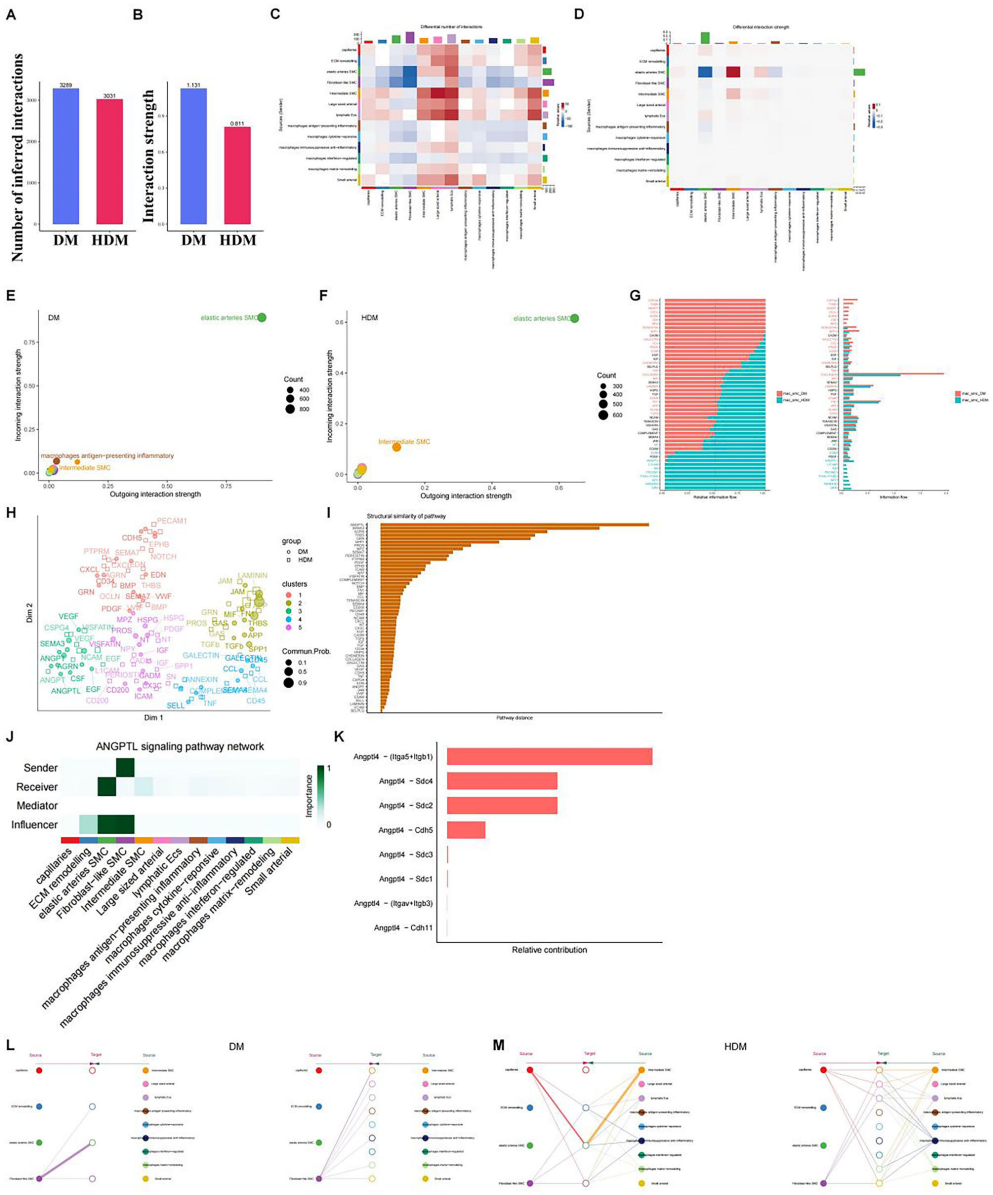
Intercellular communication changes in HDM aortic tissues. A) Total number of ligand‐receptor interactions in aortic tissue subpopulations in DM and HDM groups. B) Overall interaction intensity between aortic tissue subpopulations in DM and HDM. The total interaction intensity was calculated by summing the communication probabilities of all inferred interactions. C) Differences in interactions between HDM and DM subpopulations. Red and blue indicate an increase and decrease in the number of interactions, respectively. The bar chart shows the sum of changes in the number of input signals for each subgroup. Sum of changes in the number of output signals for each subpopulation (right). D) Interaction intensity between HDM and DM subpopulations. E) Intensity of input and output interactions for each subpopulation in DM. Dot size indicates the number of interactions. F) Intensity of input and output interactions for each subpopulation in HDM. Dot size indicates the number of interactions. G) Relative information flow for each signaling pathway between HDM and DM subpopulations. Information flow is the sum of communication probabilities between all subpopulation pairs. H) Joint manifolds show DM and HDM communication networks and group signal pathways based on functional similarity. A high degree of functional similarity means that the primary sender and receiver are similar. I) Learn the Euclidean distance of each pathway in the combined manifold. The greater the distance, the greater the difference in functional similarity (i.e., similarity between the sender and receiver) between DM and HDM. Only overlapping paths between DM and HDM are displayed. J) Central analysis of primary senders and receivers of ANGPTL signaling pathway networks in DM and HDM. K) Relative contribution of each ligand‐receptor in HDM to the overall signaling of the ANGPTL signaling pathway. L,M) Layered graph showing the inferred communication network for ANGPTL4 – (ITGA5 + ITGB1) signaling in DM. M, Hierarchy graph showing the inferred communication network for ANGPTL4 – (ITGA5 + ITGB1) signaling in HDM. Solid and hollow circles represent targets and sources, respectively. The width of the edges represents the intensity of the interaction, and the size of the circle is proportional to the number of nuclei in each subpopulation. The edges are color‐coded according to the signal source.

CellChat was applied to infer ligand‐receptor interactions among subpopulations of ECs, macrophages, and VSMCs in vivo for each condition based on snRNA‐seq data.^[^ ^25^ ^]^ The inferred total number (**Figure** **6**A) and strength of interactions (Figure 6B) significantly decreased in HDM, reflecting a reduced intercellular communication under hypoglycemia conditions, consistent with reports associated with PCD.^[^ ^10^ ^]^ The number (Figure 6C) and strength (Figure 6D) of the interactions for outgoing and incoming signals significantly increased in VSMC and endothelial subpopulations, confirming their central roles in the pathological changes of HDM. Macrophages, particularly the proinflammatory subpopulations, such as antigen‐presenting inflammatory, cytokine‐responsive, and IFN‐regulated macrophages, demonstrated increased communication with the inflammatory endothelial subpopulation, such as lymphatic ECs. Chronic inflammation and fibrosis‐related macrophages, such as immunosuppressive anti‐inflammation and matrix remodeling, also increased communication with large‐sized arterial ECs. Comparing the relative positions of VSMCs in the 2D signal space between DM (Figure 6E) and HDM (Figure 6F) suggests a substantial change in intercellular communication.

Next, this study compared the relative information flow of each signaling pathway in DM versus HDM (Figure 6G) and identified pathways that significantly increase signaling in HDM (e.g., ANGPTL, PDGF, ICAM, NT, JAM, and SEMA) or specific pathways (e.g., L1CAM, SN NPY, GRN, ITGL‐ITGB2, and ANNXIN). The signaling pathways are grouped according to their functional similarity (i.e., similarity of the sender and receiver) by performing joint manifold learning on the inferred communication network (Figure 6H). The change in functional similarity between the two conditions is reflected by learning the Euclidean distance of each pathway in the combined manifold.^[^ ^28^ ^]^ As shown, the distance of the ANGPTL pathway was the greatest (Figure 6I). Network centrality analysis confirmed that in the HDM state, the middle SMC subset was at the top of the signal sender, mediator, and influencer of the ANGPTL pathway; elastic artery SMCs play the role of the highest signal receiver and influencer; and fibroblast‐like SMCs play the role of the highest signal sender and influencer (Figure 6J). Next, ANGPTL – ITGA5 + ITGN1 was the ligand‐receptor pair that mostly contributed to the ANGPTL signaling network in thoracic aorta tissues of HDM mice (Figure 6K). ANGPTL – (ITGA5 + ITGN1) signaling was enhanced in HCM. The paracrine signal of ANGPTL – (ITGA5 + ITGN1) signaling was improved in HCM. The paracrine signal of ANGPTL received by fibroblast‐like SMCs was mainly secreted by effector intermediate SMCs, lymphatic ECs, immunosuppressive anti‐inflammatory macrophages, and matrix‐remodeling macrophages (Figure 6L,M).

#### 2.4.4 ANGPTL4 Silencing Alleviates Fibrosis in Diabetic Thoracic Aorta Under Hypoglycemia

**Figure 7:**
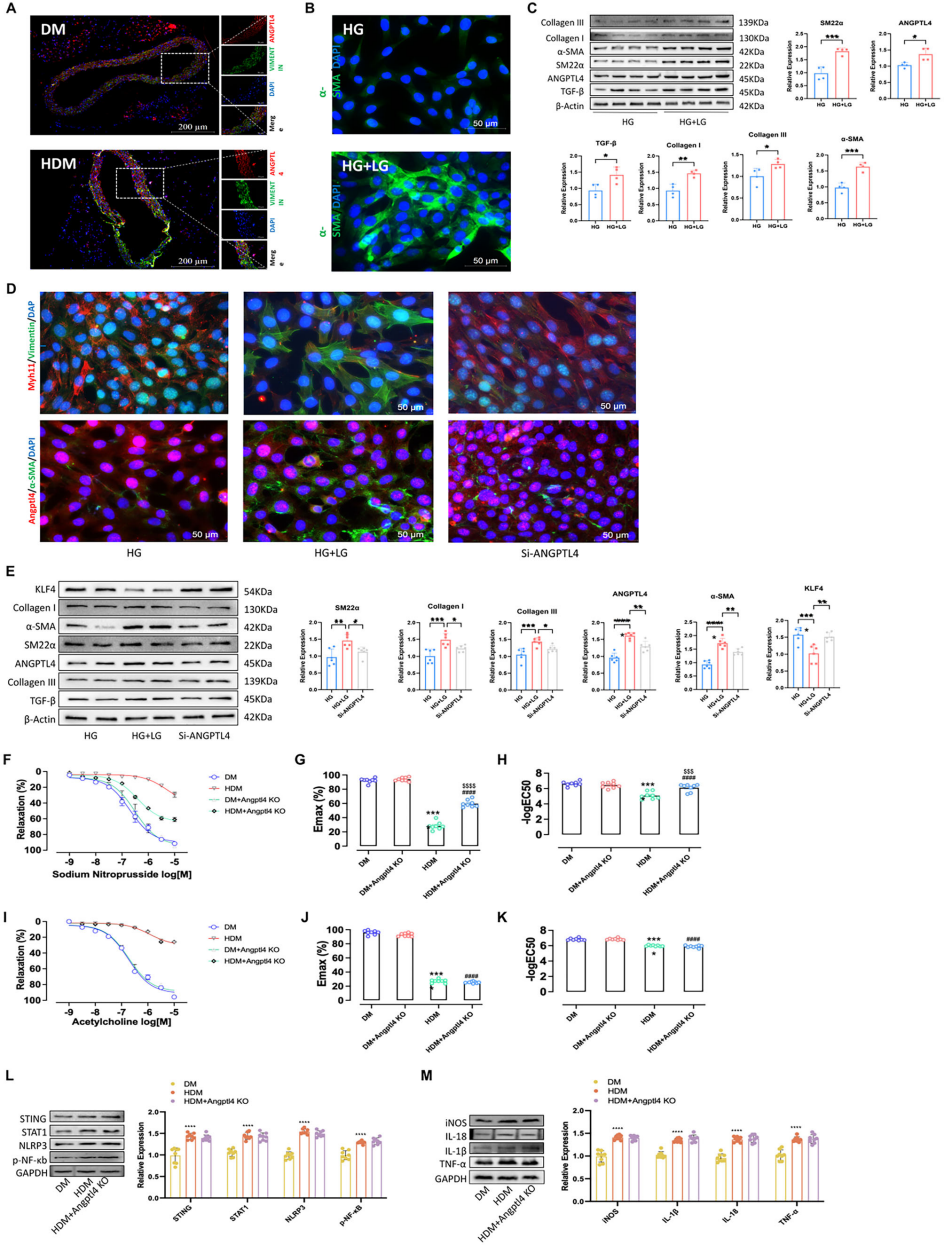
ANGPTL4 functions as a transcription activator in fibrosis under hypoglycemia conditions. A) Immunostaining confirmed ANGPTL4 and VIMENTIN expression in aortic tissues from DM (top) and HDM (bottom). Scale bar, 200 µm. B) Immunostaining of α‐SMA showing the fibrosis caused by low glucose treatment with high‐glucose cultured VSMCs. Scale bar, 50 µm. C) Immunoblotting showing the protein expression changes in ANGPTL4 and representative fibrosis markers, such as COLLAGEN I, COLLAGEN III, α‐SMA, SM22α, ANGPTL4, and TGF‐β, in the HG group (n = 8) and HG + LG group (n = 8). *P < 0.05; **P < 0.01, Wilcoxon rank‐sum test. D) Immunostaining showing the changes in protein expression of α‐SMA and the representative VSMC marker MYH11 by ANGPTL4 knockdown in HG and HG + LG groups. E) Immunoblotting showing the protein expression changes in KLF4, COLLAGEN I, α‐SMA, SM22α, ANGPTL4, COLLAGEN III, and TGF‐β by ANGPTL4 knockdown in the HG group (n = 8) and HG + LG group (n = 8). *P < 0.05; **P < 0.01, Wilcoxon rank‐sum test. F–H) Left, Vascular smooth muscle vasodilation in DM, HDM, DM + Angptl4 KO, and HDM + Angptl4 KO endothelium‐free thoracic aorta rings (n = 8). Angptl4 KO has an effect to attenuate the curve of vascular smooth muscle vasodilation. Angptl4 KO has an effect to attenuate the curve of vascular smooth muscle vasodilation. Right, EC_50_ of endothelium‐free thoracic aortic rings in the above group. Angptl4 KO has an effect to attenuate the EC_50_ and maximum diastolic rate of the thoracic aorta under hypoglycemia. ^****^ *P* < 0.0001, HDM versus DM, ^####^DM + Angptl4 KO versus HDM + Angptl4 KO; *P* < 0.0001, ^$$$$^HDM versus HDM + Angptl4 KO; *P* < 0.0001, Wilcoxon rank‐sum test. I–K) Left, Endothelium‐dependent vasodilation in DM, HDM, DM + Angptl4 KO, and HDM + Angptl4 KO thoracic aorta rings (n = 8). Angptl4 KO has no effect on attenuating the curve of endothelium‐dependent vasodilation. Right, EC_50_ of thoracic aortic rings in the above group. Angptl4 KO has no effect to attenuate the EC_50_ and maximum diastolic rate of the thoracic aorta under hypoglycemia. ^****^ *P* < 0.0001, HDM versus DM, ^####^DM + Angptl4 KO versus HDM + Angptl4 KO; *P* < 0.0001, Wilcoxon rank‐sum test. L,M) Western blot assays confirming the significant change of macrophages proinflammatory polarization markers and their signaling pathways in thoracic aorta tissues from HDM (n = 8) compared to DM mice (n = 8). ^****^ *P* < 0.0001, HDM + Angptl4 KO(n = 8) compared to HDM (n = 8). *P* = ns, Wilcoxon rank‐sum test.

Immunostaining confirmed that the protein levels of ANGPTL4 and fibrosis marker VIMENTIN significantly increased in thoracic aorta tissues, including EC and VSMC, in the HDM group (**Figure** **7**A). In vitro immunostaining showed significantly increased expression of the fibrosis marker α‐SMA in the HG + LG group, and immunoblotting further confirmed higher protein levels of fibrosis markers, such as COLLAGEN I, COLLAGEN III, α‐SMA, SM22α, ANGPTL4, and transforming growth factor‐β (TGF‐β), in the HG + LG group (n = 8) than in the HG group (n = 8; *P* < 0.05, Wilcoxon rank‐sum test; Figure 7B,C). To explore the role of ANGPTL4 in activating aortic VSMCs in diabetes under hypoglycemia, small interfering RNA (siRNA)‐mediated silencing of ANGPTL4 was conducted in HDM mouse aortic SMCs. Immunostaining showed that silencing ANGPTL4 expression reduced fibrosis induced by hypoglycemia and treated in high‐glucose cultured VSMCs (Figure 7D). Immunoblotting further confirmed that silencing of ANGPTL4 expression attenuates the expression levels of low glucose‐induced fibrosis markers, such as KLF4, COLLAGEN I, α‐SMA, SM22α, ANGPTL4, COLLAGEN III, and TGF‐β (Figure 7E).

In addition, we applied Angptl4‐KO mice in vivo to further confirm the role of ANGPTL4 as an activator in the fibrosis of diabetic thoracic aorta under hypoglycemia. In the endothelium‐free thoracic aorta rings, relaxant responses to sodium nitroprusside (SNP) in aortic segments of insulin‐treated HDM mice were significantly higher than those in the DM group, whereas hypoglycemia in these vessels increased the median effective dose (EC_50_) and maximum diastolic rate. It is worth noting that impairment of vascular smooth muscle relaxation of thoracic aortas induced by hypoglycemia revealed a marked attenuate in Angptl4‐KO diabetic mice, and hypoglycaemia in these vessels elicited an increase in EC_50_ also has been significantly alleviated in Angptl4‐KO diabetic mice (Figure 7F–H). Meanwhile, relaxant responses to acetylcholine (Ach) in aortic segments of insulin‐treated HDM mice were significantly higher than those in the DM group, whereas hypoglycemia in these vessels increased the EC_50_ and maximum diastolic rate. This phenomenon is also observed in diabetic Angptl4‐KO and insulin‐treated diabetic Angptl4‐KO mice. Importantly, there has no significant difference of the relaxant responses to acetylcholine (Ach) and EC_50_ between HDM and insulin‐treated diabetic Angptl4‐KO mice (Figure 3I–K), which means ANGPTL4 knockout exerts no influence on hypoglycemia‐induced impairment of endothelial dependent vasodilation. Previous researchers have also detected this phenomenon in diabetic Angptl4‐KO mice (Figure 7I–K). Furthermore, the immunoblotting results indicated that in the hypoglycemic Angptl4‐KO mice, the increased expression of markers of macrophage inflammatory polarization like iNOS and TNF‐α, and its signaling pathway like STING, STAT1, and NF‐κβ/NLRP3 in aorta was not suppressed (Figure 7L,M). These results suggested that the silencing of ANGPTL4 can ttenuate the activation of VSMC fibrosis in the thoracic aorta of HDM, implying that ANGPTL4 might function as a transcription activator in fibrosis under hypoglycemia conditions.

## 3 Discussion

Exploring the lineage‐specific regulatory changes of thoracic aortic tissues under hypoglycemia conditions is important in understanding the damage of hypoglycemia in aging diabetic patients. This study first presented a panoramic picture of hypoglycemic effects on the thoracic aorta in a diabetes rodent animal model. These effects include ECs PANoptosis, macrophage proinflammatory polarization, VSMC fibrosis, and ANGPTL4‐mediated intercellular communication. Under hypoglycemia, these changes induce endothelial cell dysfunction in diabetes, resulting in cardiovascular disorders. The latter types are caused by multiple lineages, where ECs are the major determinant of endothelial cell inflammatory PANoptosis.

To the best of our knowledge, macrovascular‐like thoracic aorta in *db/db* mice has been divided into subpopulations of small arterial ECs, large‐sized arterial ECs, capillaries, and lymphatic ECs, with a significant increase in the relative proportion of lymphatic ECs and a decrease in the relative proportion of large‐sized arterial ECs. The bioinformatics analysis results align with previous findings that hypoglycemia, which causes EC inflammatory PANoptosis in the thoracic aorta of diabetic rodent animal models, is the main reason for endothelial cell dysfunction. Our data explain previous observations that hypoglycemia results in diffuse endothelial cell dysfunction and a proinflammatory, proatherothrombotic, and procoagulant state, which are generated from PANoptosized ECs in T2DM with standard treatment.^[^ ^8^, ^9^ ^]^ PANoptosis, which is modulated by diverse PANoptosomes, including ZBP1‐PANoptosome, AIM2‐PANoptosome, and RIPK1‐PANoptosome complexes, with assembly by pyroptotic, apoptotic, and necroptotic mediators, exerts essential roles in various infectious and inflammatory diseases and cancers.^[^ ^35^ ^]^ The role of PANoptosome in ECs of HDM remains elusive. ZBP1 senses double‐stranded RNA, AIM2 senses double‐stranded DNA, and RIPK senses Yersinia. These activators of PANoptosome sensors have no direct relationship with hypoglycemia. Hence, the modality of sensing of PANoptosome to hypoglycemia in diabetic ECs should be experimentally explored. The functional enrichment analysis reflected the features known for inflammatory PCD and endothelial dysfunction, such as the AGE‐RAGE signaling pathway, the Renin secretion pathway, the GnRH signaling pathway, and elastic fiber formation. Some genes greatly changed in DRN centrality in HDM have been implicated in cell death or inflammation. ETS2, which has been reported to determine the inflammatory state of ECs in advanced atherosclerotic lesions, was significantly increased in HDM.^[^ ^29^ ^]^ NR4A3, associated with fibrosis upregulation, vascular regeneration inhibition, and inflammation activation, decreased in HDM.^[^ ^30^ ^]^ These changes indicate that hypoglycemia can attenuate protective effects and aggravate HDM injury to ECs.

Macrophage polarization is different between M1 and M2 subpopulations. First, macrophages have been grouped into five subpopulations in DM and HDM, with a significant increase in the relative proportion of cytokine‐responsive macrophages in HDM, indicating proinflammatory polarization of macrophages, which is also proven by immunofluorescence staining in vivo. Consistent with previous studies, these phenomena explain how hypoglycemia causes oxidative stress and increases iNOS levels in the diabetic animal model. Interestingly, the decrease in the relative proportion of antigen‐presenting macrophages in HDM does not represent a decrease in immune or inflammatory response. There are three types of antigen‐presenting cells (APC): major histocompatibility complex (MHC) class I presentation, MHC class II presentation, and cross‐presentation.^[^ ^62^, ^63^ ^]^ MHC class I presents the endogenous proteins that have no relationship with hypoglycemia. MHC class II presents exogenous proteins, such as bacteria, parasites, antigen‐antibody complexes, and dead cells, which are taken up by APCs through endocytosis. Cross‐presentation is favored by DCs independent of ECs. Hence, it is speculated that antigen‐presenting macrophages in HDM are MHC class II‐presenting. Therefore, with EC PANoptosis in HDM, fewer dead cells are detected by decreased exogenous proteins. The functional enrichment analysis accurately reflects the features known for macrophage proinflammatory polarization, such as amoebiasis, *Salmonella* infection, leukocyte chemotaxis, leukocyte migration, and endocytosis‐related signaling pathways. Some genes highly changed in DRN centrality in HDM have been implicated in cell death or inflammation, macrophage proinflammatory polarization. Based on multiple evidence from independent analyses, a list of potential key genes in the transition of macrophages to an activated state was obtained, such as C3 and ADGRE5, which are significantly upregulated on the macrophage activation trajectory, with a significant expression increase in HDM. Recently, inhibiting the activation and deposition of complement C3 hampers the phagocytosis of macrophages to cellular debris, leading to its accumulation in arterial vessel walls and accelerating atherosclerosis development.^[^ ^45^ ^]^ ADGRE5 is important in cell adhesion, migration, proliferation, inflammatory response, and tumor development.^[^ ^46^ ^]^ The key genes in this study reflect the proinflammatory polarization of macrophages in diabetic macrovascular ECs under hypoglycemia conditions.

The elastic artery SMCs expressed high levels of fibrotic marker genes, such as MFAP4, and inflammatory response markers, such as MT1, which were significantly increased in HDM compared to DM. The SMCs expressed high levels of SORBS2, which had a negative correlation with atherosclerosis and were significantly decreased in HDM compared to DM. This is the first report that showed genes related to inflammation are associated with hypoglycemia in the thoracic aorta of a diabetic rodent animal model. These genes contributed to hypoglycemia‐mediated VSMC fibrosis via chronic inflammation. Further, functional enrichment analysis revealed the features known for SMC fibrosis and chronic inflammation, such as ECM remodeling, ECM, and the Apelin signaling pathway. These bioinformatics analysis results were in line with new findings regarding hypoglycemia that causes VSMC fibrosis in diabetic animals. Some genes greatly changed in DRN centrality in HDM have been implicated in fibrosis and chronic inflammation. Based on the above‐mentioned independent analyses, a list of potential key genes in the transition of VSMCs to a fibrosis condition was obtained, such as *Klf2* and *Nr4a1*. The TF KLF2‐FOXP1 regulatory network is important in regulating the activation of the NLRP3 inflammatory somatic complex and controlling the occurrence and development of atherosclerosis.^[^ ^58^ ^]^ KLF2 expression significantly decreased in HDM, indicating that hypoglycemia attenuates the protective effects in diabetic vessels. Besides, NR4A1 has been recognized as an activator of a T cell‐induced immune response that can induce fibrosis through TGF‐β signaling both in vitro and in vivo.^[^ ^58^, ^59^, ^63^, ^64^ ^]^ NR4A1 expression decreased in HDM, and its precise role in VSMC fibrosis needs to be experimentally determined.

New evidence suggests that intercellular interaction among ECs, macrophages, and VSMCs is essential to cardiovascular diseases.^[^ ^15^ ^]^ Results about the number and strength of interactions in hypoglycemia are lethal for the cardiovascular system, but also further reveal that VSMCs coordinate the responses of fibrosis, which belongs to the end stage of inflammation caused by endothelial cell inflammatory PANoptosis under hypoglycemia. Endothelial cell dysfunction has a close relationship with ANGPTL4.^[^ ^65^ ^]^ However, these studies focused on oncology, lipid metabolism, and atherosclerosis, and there is a continuous lack of research in the area of hypoglycemia‐related cardiovascular events, although ANGPTL4 has been first reported as a novel hypothalamic hypoglycemia‐induced gene in 2001.^[^ ^65^ ^]^ ANGPTL4 has a close relationship with VSMC fibrosis under hypoglycemia, which might explain its potential role in glucose metabolism in DM. The possible reason behind this relationship is that in hypoglycemia, increased ANGPTL4 levels induces fibrosis in the liver, adipose tissue, or muscles, resulting in glucose metabolism dysfunction.

**Figure 8:**
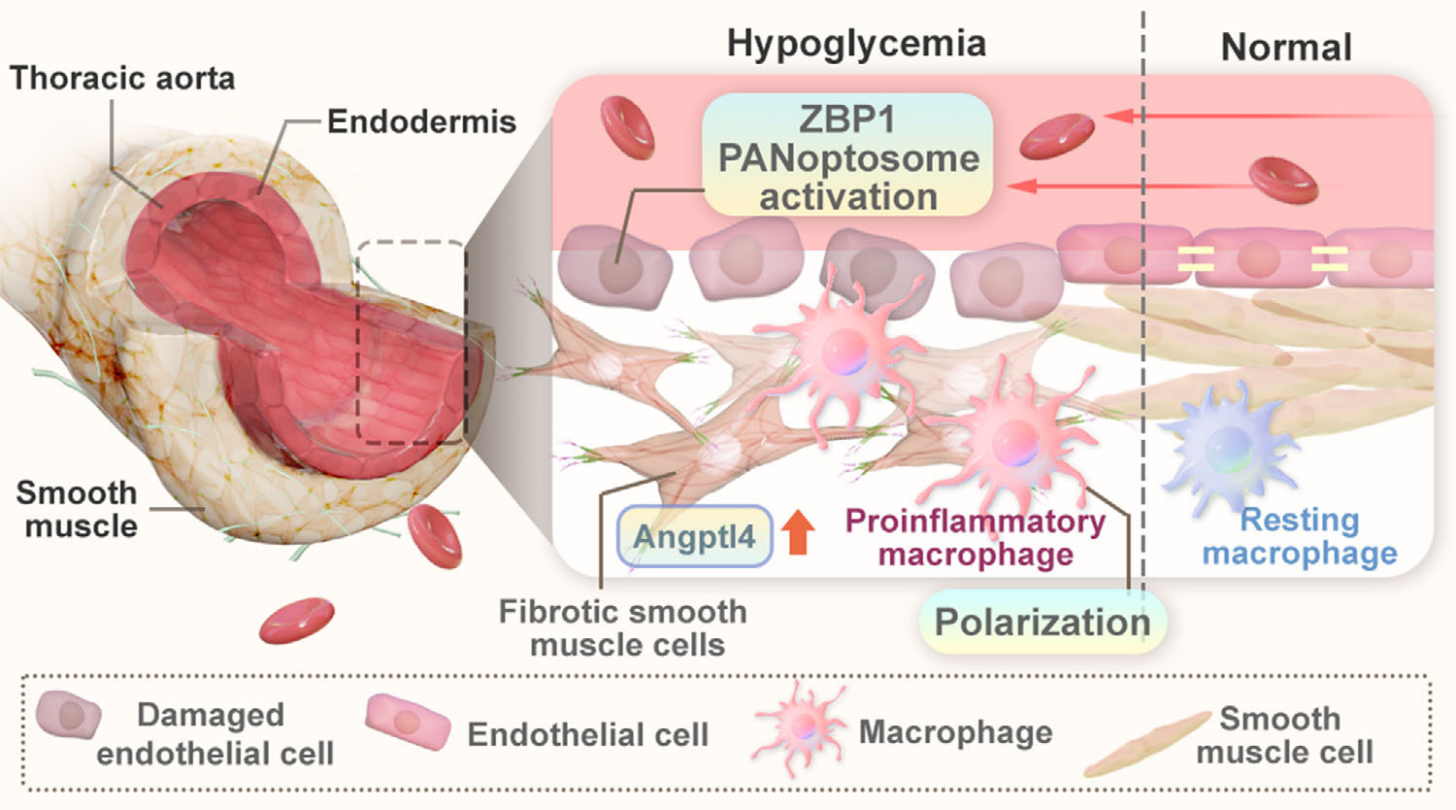
The schematic illustration on hypoglycemia induces PANoptosis of endothelial cells, proinflammatory polarization of macrophages, and fibrosis of vascular smooth muscle cells (VSMC), thereby contributing to diabetic macro‐vascular dysfunction. Hypoglycemia triggers endothelial cell PANoptosis by activating the ZBP1‐mediated PANoptosome. The inflammatory factors (e.g., IL‐1β and IL‐18) released during PANoptosis drive macrophages toward proinflammatory polarization, while the activation of the ANGPTL4 signaling pathway induces vascular smooth muscle cell (VSMC) fibrosis, ultimately leading to diabetic macrovascular dysfunction.

In summary, this study contributed to a comprehensive analysis of lineage‐specific regulatory changes in HDM. We identified potential genes that are associated with the transition to an inflammatory PCD state of ECs, proinflammatory polarization of macrophages, and fibrosis of VSMCs. This study revealed experimental evidence supporting that ABGPTL4 functions as a transcription activator in vascular fibrosis (**Figure** **8**).

## 4 Experimental Section

### Diabetic Hypoglycemia Model

Fifty‐week‐old male *db/db* DM were purchased from Hangzhou Ziyuan Laboratory Animal Technology Co., Ltd. All animal procedures followed the China Animal Protection Law guidelines and were approved by the Institutional Ethics Committee of Chongqing Medical University and the State Science and Technology Commission of China. Animals were exposed to a 12 h light‐dark cycle at 22 to 25 °C with food and water access ad libitum. The mice were randomly divided into two groups (n = 8): DM and HDM. The construction of the HDM model followed the experimental design of a previous study by Luo et al^[^ ^10^ ^]^ with slight modification.

For the construction of the HDM model, 10.0 U kg^−1^ insulin was injected into DM mice after one night fasting to provoke severe hypoglycemia. Daily 1 h hypoglycemic episodes were sustained over 5 consecutive days. Blood glucose was monitored by puncture every 30 min to ensure that the mouse blood glucose was maintained in an IHSG level 2 standard.^[^ ^4^ ^]^ Mice were given 50% glucose in phosphate‐buffered saline (PBS) to stop hypoglycemic episodes. No mice showed seizures or comas during hypoglycemic episodes. Mice were sacrificed with pentobarbital sodium anesthesia, and blood was drawn from the aorta for subsequent experiments.

### Diabetic and Diabetic Hypoglycemic Model of Angptl4‐KO Mice

Male 8‐week‐old Angptl4‐KO mice were purchased from Chongqing Zhang'an Technology Co., Ltd. All animal procedures followed the China Animal Protection Law guidelines and were approved by the Institutional Ethics Committee of Chongqing Medical University and the State Science and Technology Commission of China. The husbandry environment of Angptl4‐KO mice was the same as db/db. Mice were treated with a high fat diet (HFD; Trophic Animal Feed High‐Tech Co, Ltd., Nantong, China) for 4 weeks, followed by administration of an intraperitoneal injection of 35 mg kg^−1^ STZ (Sigma, Saint Louis, MO, USA) in citric acid solution (pH 4.2–4.5) (Beyotime Shanghai, China) according to their fasting weights. Citrate buffer (vehicle) alone was injected into normal control rats (control group). After 4 weeks of administering STZ injection, mice with non‐fasting plasma glucose >16.7 mM were deemed as diabetic.^[^ ^5^ ^]^ The experimental grouping for the construction of the HDM model was the same as that of db/db mouse.

### Thoracic Aorta Collection and Cell Isolation

Mice were placed in an anesthesia machine using the isoflurane anesthesia method to ensure a pain‐free state. Upon dissecting the thoracic cavity, the thoracic aorta and surrounding tissues were carefully separated using a microsurgical instrument. The extracted thoracic aortic tissues were cryopreserved. Frozen aortic tissues were thawed, dissected into small pieces, and washed once with cold PBS (20 012 050, Gibco). Tissue slices were transferred to a 50‐mL Falcon tube containing 30 mL lysis buffer [0.32 M sucrose, 5 mM CaCl_2_, 3 mM C_4_H_6_MgO_4_, 0.5 mM EGTA, 10 mM Tris‐HCl 8.0, 2 mM EDTA, 1 mM phenylmethylsulfonyl fluoride (PMSF), 1 mM dithiothreitol (DTT), and 80 U mL^−1^ RI]. Tissues were mixed with a T‐25 Ultra‐Turrax homogenization probe (IKA) at 24,000 rpm for 15 s. The mixture was evenly homogenized with a glass straw (40 mL) and a tight stirring rod 10 times. After 10 min incubation on ice, the crude nucleus suspension was passed through 100 and 70 µm nylon mesh cell filters (BD Biosciences), followed by 10 min centrifugation (700 × *g*, 4 °C). The supernatant was cautiously eliminated, and the isolated crude nuclei were suspended in 30 mL sucrose buffer (2.1 M sucrose, 3 mM C_4_H_6_MgO_4_, 10 mM Tris‐HCl 8.0, 1 mM PMSF, 1 mM DTT, and 80 U mL^−1^ RI) and centrifuged at 4 °C (Beckman Avanti S‐25; 13,000 rpm, 60 min). Again, the supernatant was eliminated, and the nucleospheres were dissolved in 10 mL nuclear suspension buffer [1% bovine serum albumin (BSA) and 200 U mL^−1^ RI in PBS] and centrifuged at 4 °C (500 × *g*, 10 min). Nuclear particles were suspended in a nuclear suspension buffer (1 mL) for additional experimentation. All procedures were performed at 4 °C. RNA inhibitors (80 U mL^−1^; Takara) were added to all buffers.

### Library Preparation for scRNA‐seq

Single‐cell transcriptome library preparation and sequencing were completed using the Bio‐Cyto platform (Biomedicine, Chongqing, China). After the preparation and quality inspection of the cell suspension, cells were added to the chip storage pool. The CytoG100 single‐cell microdroplet generation instrument (Biomedicine) was used to achieve high‐throughput cell capture using microfluidics, droplet encapsulation, and barcode labeling, forming a microdroplet system. Gel beads released probes to bind with mRNA released from cells, followed by cDNA synthesis, polymerase chain reaction amplification, and quality control. The construction of single‐cell libraries followed the operation of the single‐cell library construction reagent kit (Bio‐Cyto G100 Library Construction Kit; Biomedicine). scRNA‐seq analysis was performed using the BGISEQ‐500 sequencing platform (BGI Genomics Co., Ltd., Shenzhen, China).

### Preprocessing and Quality Control of scRNA‐seq Data

To determine exon and intron readings captured using scRNA‐seq, a protocol for the customized “pre‐mRNA” reference package 10x Genomics was designed based on the human reference genome dataset (version refdata‐geo‐grch38‐2020‐a), and the original sequencing readings were aligned with the “pre‐mRNA” reference sequence in the official toolkit Cell Ranger (version 4.0.0). For additional data preprocessing, the output nuclear gene expression matrix was imported into Seurat (version 3.2.3). Genes counting less than three nuclei were filtered out to exclude genes that might have been detected due to random noise. Nuclei were counted for their unique molecular identifier (UMI; 500 < nCount_RNA < 50000), genes (300 < nFeature_RNA < 7000), the proportion of mitochondrial genes (%Mito < 0.05), and ribosomal gene ratio (%Ribo < 0.05). Inferior nuclei that may be caused by heavy states or other technical noise were removed. To remove potential  γ states, a γ score > 0.35 predicted by Scrublet47 was filtered out. For further analysis, nuclei expressing multiple lineage marker genes were excluded.

### Normalization, Feature Selection, Integration, Scaling, and Clustering of scRNA‐seq Data

Seurat in R version 4.4.0 was used to proceed with the analysis. For each sample, the sum of UMI counts for each core was normalized to 10000 and log‐transformed. Using Seurat's FindVariableFeatures function, 2000 genes were selected for each sample. Cells from all samples were harmonized to correct potential batch effects and identify shared cellular states between samples. These were also standardized using the “ScaleData” function. Data were linearly dimensionality‐reduced by principal component analysis (PCA). Using the first 16 PCA components, the shared nearest‐neighbor graph of the kernel was calculated. SNN diagrams were 2D using a nonlinear dimensionality reduction approach (UMAP). Louvain algorithm was applied for clustering.

### Identification of DEGs in a Specific Cell Type Based on scRNA‐seq Data

Seurat's FindMarkers function detected the differential expression of DM and HDM. Wilcoxon rank‐sum test was applied to detect significant differences in gene expression with the following criteria: mean expression log_2_FC > 0.3, *P* < 0.05, based on Bonferroni correction.

### Pseudo‐Bulk RNA‐seq Analysis

For each cell type, the raw UMI count matrix of scRNA‐seq data for each gene for each sample was summed to a pseudo‐batch RNA‐seq dataset. By default, the pseudo‐bulk RNA‐seq dataset was analyzed for differential expression using the R package DESeq2. Statistical significance was set to *P* < 0.05 after multiple test adjustments.

### Gene Set Enrichment Analysis (GSEA) Based on scRNA‐Seq Data

Before GSEA, all genes expressed in scRNA‐seq data were presorted by Signal2Noise [the difference between the mean of DM and HDM scaled by standard deviation (SD)]. The ordered gene list was imported into GSEA version 4.0.1. *P* < 0.05 was considered statistically significant. This analysis used the MSigDB (version 7.2) precompiled set of typical pathway genes (“c2.cp”).

### Differential GRN Analysis Based on scRNA‐Seq Data

A specific lineage of GRNs was constructed based on a single‐core dataset, and GRNs between DM and HDM were compared and analyzed using the method implemented in bigScale214. Briefly, the GRN of a particular lineage was inferred separately for each condition using the “computer network” function. The “Homogenize.Networks” function was used to homogenize the sides of an inferred GRN across the network. Changes in the node centrality (relative importance of genes in the network) of DM versus HDM were determined by “comparison.” The gene ranking based on central variation was the output of each of the four central measures (degree, intermediate degree, proximity, and pagerank). Cytoscape (version 3.7.1) was applied for the visualization of networks.

### Trajectory Inference Based on scRNA‐Seq Data

By default, trajectory inference was applied using Slingshot (version 1.4.0), ordering the nuclei along biological processes of interest, such as fibroblast activation. Combined scRNA‐seq data for DM and HDM were thought to increase the robustness of inference. The Kolmogorov‐Smirnov test was used to evaluate whether there was a difference in the pseudo‐time distribution between the two scenarios. Subsequently, genes with different expression patterns on the trajectory between the two conditions were identified using tradeSeq (version 1.6.0). For each case, a negative binomial generalized additive model (function “fitGAM”, nknots = 5) was applied to estimate the smooth expression profile of each gene along the inferred trajectory. The fitted model acted as an input to the “conditionTest” function to test whether genes exhibit different expression patterns under different conditions (known as differential expression pattern analysis). The significance threshold was set to *P* < 0.05 after multiple test adjustments.

### Ligand‐Receptor Interaction Analysis Based on scRNA‐Seq Data

Ligand–receptor interactions between subpopulations under each condition were inferred separately using CellChat (version 0.5.5), and signal changes in HDM were determined by performing comparative analysis through software tutorials. After detecting overexpressed ligands or receptors in each subpopulation, any potential association between two subpopulations was quantified using a communication probability (interaction intensity) value modeled by the law of mass action. Significant interactions (*P* < 0.05) were determined by randomly arranging the subpopulation tags and recalculating the permutation test of communication probabilities. The main input and output signal patterns for each subpopulation in DM and HDM were detected using pattern recognition methods. Through a network‐centric analysis, the main signal sources and targets of a specific path signal network were inferred. Joint manifold learning was performed on the communication networks of DM and HDM, and signal pathways were grouped according to functional similarity (high similarity means that the main sender and receiver are the same). According to the Euclidean distance in the acquired joint manifold, a signal pathway with significant changes in functional similarity between HDM and DM was identified. Comparing the overall information flow of HDM to each signaling pathway could identify conserved or highly variable signaling pathways in HDM.

### Preparation of Protein Samples from Mouse Thoracic Aortas

The thoracic aorta of mice was rinsed with sterile PBS buffer to remove blood and other impurities. The washed aorta was placed on ice and chopped finely with a tissue homogenizer. Tissue blocks were added to the tubes containing phosphate buffer (pH 7.4) and protease inhibitors and homogenized using a tissue homogenizer. The supernatant obtained by centrifuging the homogenate was ultrasonically broken using an ultrasonic breaker to make the protein more homogeneous. The crushed supernatant was centrifuged to remove the precipitate. Protein samples were divided into sterile centrifuge tubes and stored in a freezer at −80 °C to avoid protein degradation.

### Vascular Reactivity in Isolated Thoracic Aortas

Fresh thoracic aortas were immediately preserved in a physiological saline solution (PSS) buffer which containing (mM): NaCl, 130; NaHCO3, 14.9; KCl, 4.7; KH2PO4, 1.18; MgSO4‐7 H2O, 2.41; CaCl2, 1.6; ethylene diamine tetraacetic acid (EDTA), 0.034; glucose, 5.5. The adhering tissues were carefully removed, and the aortas were cut into 3 mm rings. To avoid any potential damage to the vascular endothelium, these rings were transferred to the Multi Myograph System (DMT620, Denmark), warmed (37 °C), and oxygenated (95% O_2_ and 5% CO_2_) to ensure optimal conditions. Vascular tension was continuously monitored using LabChart software (DMT620). The aortic rings were allowed to equilibrate for 90 min at an initial tension of 0.5 g. ACh (10^−9^‒10^−5^) was added for endothelium‐dependent relaxation^[^ ^53^ ^]^ and PE, and concentration‐response curves were plotted. Vascular function was reported at EC_50_, and E_max_ and logEC_50_ were determined by nonlinear regression analysis using GraphPad version 9.0.

The apoptosis inhibitor Z‐VAD‐FMK (10 mg kg^−1^) and the pyroptosis inhibitor disulfiram (50 mg kg^−1^) were administrated during hypoglycemia with intraperitoneal injection. The necroptosis inhibitor bardoxolone (10 mg kg^−1^) was added to drinking water during hypoglycemia.

### Vascular Smooth Muscle Function in Isolated Thoracic Aortas

For the preparation of endothelial‐free vascular rings, filter paper strips were used to rub the interior of the vascular ring for 1 min after preserved thoracic aortas in PSS solution buffer.^[^ ^65^ ^]^ After ruined the endothelium of thoracic aortas, the disposition of adhering tissues and vascular rings was the same as described.

Vascular smooth muscle was continuously monitored using LabChart software (DMT620). The aortic rings were allowed to equilibrate for 90 min at an initial tension of 0.5 g. Sodium nitroprusside (10^−9^‒10^−5^ M) was added for endothelium‐dependent relaxation and PE, and concentration‐response curves were plotted. Vascular smooth muscle function was reported at EC_50_, and Emax and logEC_50_ were determined by nonlinear regression analysis using GraphPad version 9.0. Contractile responses were assessed by the incubation of aortic segments with phenylephrine (Phe, 10^−8^ to 10^−6^ M). The diastolic function of vascular smooth muscle was analyzed by Sodium nitroprusside (SNP, 10^−8^ to 10^−4.5^ M) to segments pre‐contracted with Phe (10^−7^ to 10^−6^ M to achieve an equivalent tone between groups). The dosage and administration of apoptosis inhibitor Z‐VAD‐FMK, necroptosis inhibitor bardoxolone, and pyroptosis inhibitor disulfiram were the same as described.

### Cell Culture and Hypoglycemic (Low Glucose) Treatment—Endothelial Cell

Primary mouse aortic endothelial cells (MAEC) were purchased from Procell (Wuhan, China), The obtained MAECs were cultured in Dulbecco's Modi ed Eagle Medium (DMEM) (Invitrogen, Carlsbad, CA, USA) contain 1 g L^−1^ (5.5 mmol L^−1^) glucose supplemented with 10% (v/v) FBS (Invitrogen) and 1% (v/v) penicillin/streptomycin (PS) (Beyotime Biotechnology, Jiangsu, China) in a humidified incubator of 5% CO2 at 37 °C. Five to seven MAEC were selected for the experiments.

### Cell Culture and Hypoglycemic (Low Glucose) Treatment—Macrophage

Primary bone marrow‐derived macrophages (BMDM) were obtained by harvesting marrow from femurs of db/db and hypoglycemic mice. Lysing red blood cells with ammonium‐chloride‐potassium lysing buffer, and then culturing cells for seven days in DMEM contain 1 g L^−1^ (5.5 mmol L^−1^) glucose supplemented with 10% FBS, 1% penicillin/streptomycin, and macrophage‐colony stimulating factor (M‐CSF, 20 ng mL^−1^). All cells were regularly tested negative for mycoplasma contamination. Confluent macrophages were selected for the experiments.

### Cell Culture and Hypoglycemic (Low Glucose) Treatment—Vascular Smooth Muscle Cell

Primary VSMCs (PVSMCs) were prepared as described previously. After severing, the surrounding adipose and vascular outer membrane tissues were removed, and the aorta was separated and cut into small pieces. The obtained PVSMCs were centrifuged at 3000 rpm for 5 min, and the precipitate was resuspended in F‐12/DMEM (2 mM L‐glutamine and 100 U mL^−1^ penicillin/streptomycin) containing 20% FBS. The cultures were incubated at 37C in a compression chamber with 95% air and 5% CO2, and the medium was changed once daily. Three to five PVSMC generations were selected for the experiments.

### Cell Culture and Hypoglycemic (Low Glucose) Treatment—Hypoglycemic (Low Glucose) Treatment

The above cells were treated with high‐glucose cell culture medium, which contains 30 mM glucose for 3 days or low‐glucose cell culture medium, which contains 1 mM glucose for 1 h, and then replaced with high‐glucose medium for another 1 h. This step was repeated thrice to simulate the process of repeated hypoglycemia.

### Cell Culture and Hypoglycemic (Low Glucose) Treatment—Co‐Culture System

Before the Transwell assay, mouse aortic endothelial cells (MAEC) (Pricella, Wuhan, China) (8 × 10^4^; 5–7 passages) were cultured on six‐well plates 1 day before transfection. The next day, cells were transfected with ZBP1‐siRNA. After 48 h of transfection, the MAECs were digested and added into the Transwell lower chamber (Labselect, Beijing, China) and cultured in high‐glucose cell culture medium. Then, macrophages were seeded into the Transwell upper chamber (0.4 µm) and cultured until confluent. Then, both the upper and lower chambers were treated by low‐glucose cell culture medium, and the cells were incubated for 10 h. After 10 h of incubation, the cells in the upper and lower chambers were processed to the next experiment

### Cell Culture and Hypoglycemic (Low Glucose) Treatment—siRNA Transfection

MAEC (8 × 10^4^; 5–7 passages) were cultured on six‐well plates 1 day before transfection. The next day, cells were transfected with 50 nM mouse siRNA or 50 nM scramble siRNA as a negative control according to the manufacturer's guidelines (Ribobio). Cells were added to each well with 6 µL Dharmafect 1 transfection reagent (GE Healthcare Dharmacon). After 6 h, cells were cultured in a buffer medium containing 2% FBS. After 48 h, total RNA was isolated, and mRNA was prepared for batch RNA‐seq analysis. Proteins were extracted and determined after 72 h using Western blot analysis. siRNA sequences for AIM2, RIPK1 and ZBP1 were 5′‐GCAGUGACAAUGACUUUAATT‐3′ and 3′‐UUAAAGUCAUUGUCACUGCTT‐5′, 5′‐GGCAGAAUGAGGCUUACAATT‐3′ and 3′‐UUGUAAGCCUCAUUCUGCCTT‐5′, 5′‐GAGACAAUCUGGAGCAAAATT‐3′and 3′‐UUUUGCUCCAGAUUGUCUCTT‐5′, respectively.

PVSMCs (8 × 104; 5–7 passages) were cultured on six‐well plates 1 day before transfection. The next day, cells were transfected with 50 nM mouse ANGPTL4 siRNA or 50 nM scramble siRNA as a negative control according to the manufacturer's guidelines (Ribobio). Cells were added to each well with 6 µL Dharmafect 1 transfection reagent (GE Healthcare Dharmacon). After 6 h, cells were cultured in a buffer medium containing 2% FBS. After 48 h, total RNA was isolated, and mRNA was prepared for batch RNA‐seq analysis. Proteins were extracted and determined after 72 h using Western blot analysis. siRNA sequences were 5′‐GGGACUGCCAGGAACUCUUTT‐3′ and 3′‐AAGAGUUCCUGGCAGUCCCTT‐5′.

### Cell Culture and Hypoglycemic (Low Glucose) Treatment—Immunoblotting

Whole cells were extracted in a radioimmunoprecipitation assay buffer comprising protease and phosphatase inhibitors on ice. The nuclear/cytosol fractionation kit (Beyotime, Beijing, China) was used to obtain the nuclear and cytosolic fractions. Identical quantities of protein samples were separated in 6 to 15% sodium dodecyl sulfate‐polyacrylamide gel electrophoresis, transferred onto polyvinylidene difluoride membranes (Millipore, Billerica, MA, USA), and blocked with 5% skimmed milk for 2 h at room temperature (RT). The membranes were immunoblotted with specific primary antibodies at 4 °C overnight with slight agitation, followed by horseradish peroxidase‐conjugated secondary antibodies (Jackson Labs, USA) for 1 h at RT. Finally, the immunoreactive bands were detected using the enhanced chemiluminescence method (Amersham Imager 600, GE, USA).

### Cell Culture and Hypoglycemic (Low Glucose) Treatment—Immunofluorescence

After various treatments, cultured cells were rinsed with PBS, added with 4% paraformaldehyde, and soaked in 0.1% Triton X‐100 for 30 min. The reaction was blocked with 3% BSA for 1 h, and cells were incubated with specific primary antibodies overnight at 4 °C and with appropriate TRITC/FITC‐conjugated secondary antibodies (diluted in 1% BSA) or Bodipy (Invitrogen, Carlsbad, CA, USA) for 1 h at 37 °C. To detect phase separation, cultured macrophages were treated with 3% hexanediol for 15 s after ox‐LDL stimulation for 24 h and incubated with BRD4 and MED1 primary antibodies. Cell nuclei were counterstained with 4′,6‐diamidino‐2‐phenylindole. The sections were viewed under a confocal laser scanning microscope (LSM800, Zeiss, Oberkochen, Germany).

### Cell Culture and Hypoglycemic (Low Glucose) Treatment—Immunohistochemistry (IHC)

The thoracic aortic tissues were deparaffinized in xylene, rehydrated in ethanol, and subsequently heated in a microwave in sodium citrate to retrieve antigens. 3% peroxide in methanol was used to inhibit the activity of endogenous peroxidases. Then, the sections were blocked in PBS containing 10% goat serum. Afterward, the sections were incubated with primary antibodies of GSDMD, BAX and RIPK‐1 at 4 °C overnight followed by incubation with the corresponding secondary antibodies conjugated with HRP at room temperature for 1 h. To visualize the immunostaining, the sections were incubated in diaminobenzidine followed by a counterstain in hematoxylin. Negative control experiments were done by omitting the primary antibodies. The images were captured by a light microscope (Leica Camera, Germany).

### Statistical Analysis

Statistical analyses were performed using GraphPad Prism version 8.0.2 (GraphPad Software, Inc.). Data were presented as mean (standard error/SD). Statistical significance was determined and indicated in the figure legends: ^*^ *P* < 0.05; ^**^ *P* < 0.01; ^***^ *P* < 0.001; ^****^ *P* < 0.0001. Kolmogorov–Smirnov test was used to check the data distribution. To compare two groups for a normal distribution, a two‐tailed unpaired Student's *t*‐test was applied. If the distribution was not normal, the Wilcoxon rank‐sum test was used (*P* > 0.05). To analyze differential expression in snRNA‐seq, the hypergeometric test in DEsingle was applied.^[^ ^28^ ^]^

## Conflict of Interest

The authors declare no conflict of interest.

## Author Contributions

D.Z., Y.P., and G.Z.Z. contributed equally. D.Z., Y.P., X.L., and A.H. conceived and designed the experiments, performed the experiments, analyzed and interpreted the data, and wrote the paper. D.Z., Y.P., M.L., D.L., S.C., N.L., and Y.H. analyzed and interpreted the data. X.L. and A.H. conceived and designed the experiments, analyzed and interpreted the data, contributed reagents, materials, analysis tools or data, and wrote the paper.

## Ethics Approval and Consent to Participate

All experimental procedures conformed with the National Institutes of Health Guidelines for the Use of Laboratory Animals and were approved by the Chongqing Medical University Animal Research Committee (ethical approval number: IACUC‐CQMU‐2023‐0273).

## Supporting information



Supporting Information

## Data Availability

All data utilized in this study are included in this article, and all data supporting the findings of this study are available on reasonable request from the corresponding author.
