# Supplementary material for: Hypoglycemia Induces Diabetic Macrovascular Endothelial Dysfunction via Endothelial Cell PANoptosis, Macrophage Polarization, and VSMC Fibrosis
Source: Adv Sci (Weinh). 2025 Jul 12;12(37):e14530. doi: 10.1002/advs.202414530 (PMC12499442; doi:10.1002/advs.202414530)
Supplement: Supplementary file 1 — Supporting Information [file ADVS-12-e14530-s001.docx]

**
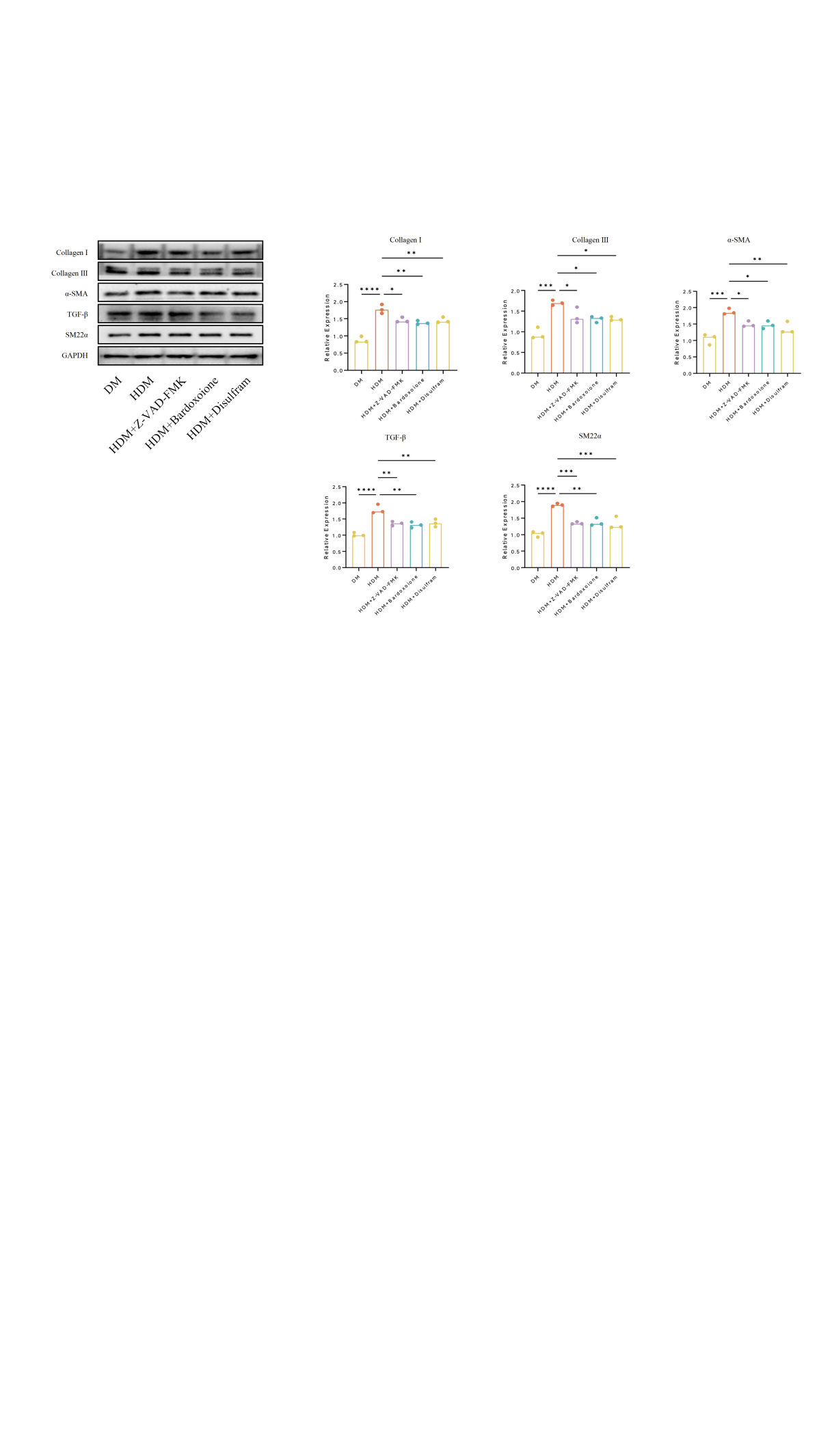
**

**Supplementary Figure S1. Z-VAD-FMK, disulfiram, and bardoxolone have no effect on the function of smooth muscle cells during hypoglycemia.**

Western blot analysis of protein levels of Collagen I, Collagen III, α-SMA, TGF-β and SM22α in the DM group (n = 8), HDM group, HDM + Z-VAD-FMK group (n = 8), HDM + bardoxolone group (n = 8) and HDM + disulfiram group (n = 8). ^*^*P* < 0.05; ***P* < 0.01; ****P* < 0.001; *****P* < 0.0001, Wilcoxon rank-sum test.


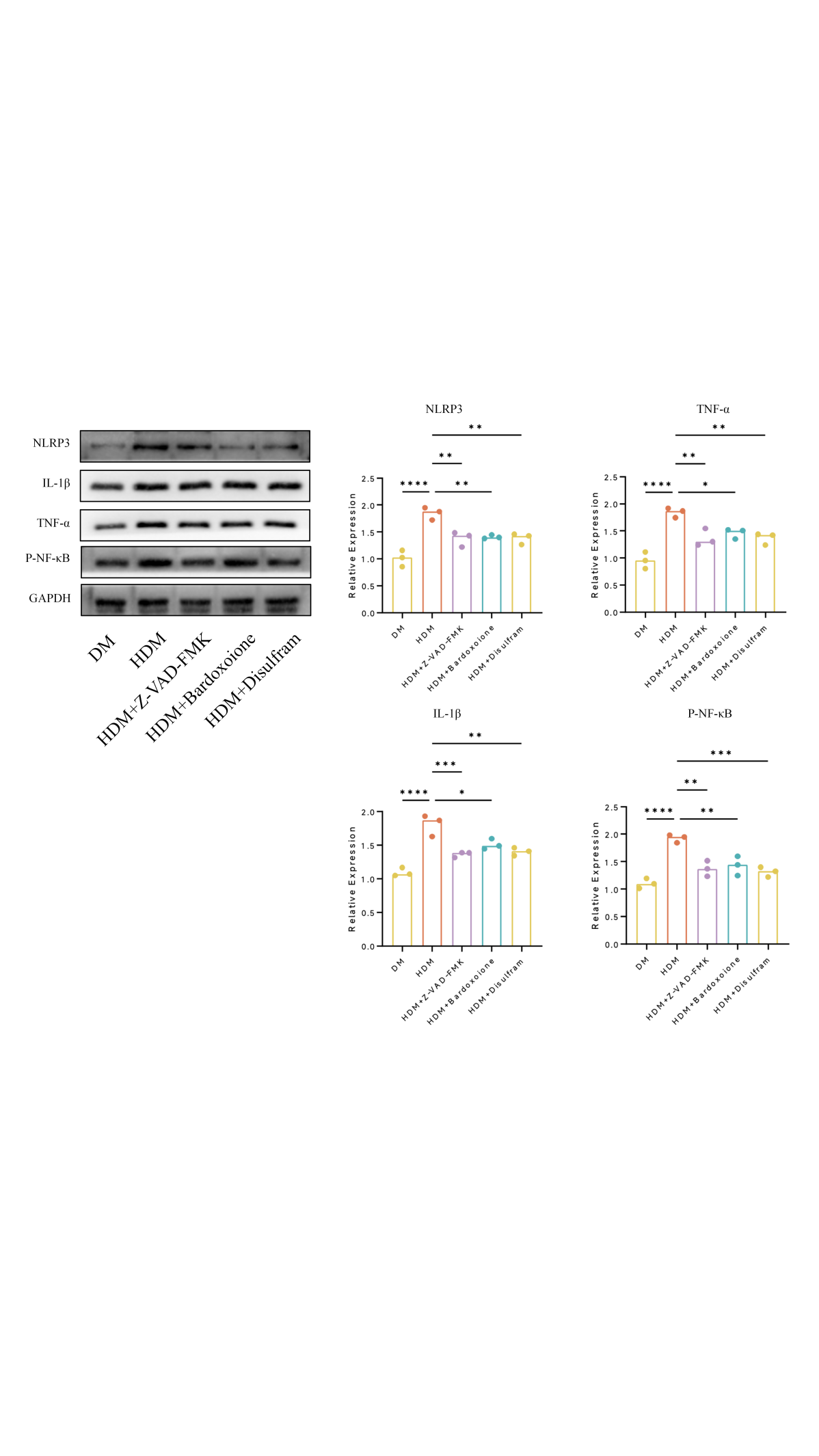


**Supplementary Figure S2. Z-VAD-FMK, disulfiram, and bardoxolone has effect to supression hypoglycemia induced macrophage inflammatory polarization.**

Western blot analysis of protein levels of NLRP3, IL-1β, TNF-α and P-NF-κB in the DM group (n = 8), HDM group, HDM + Z-VAD-FMK group (n = 8), HDM + bardoxolone group (n = 8) and HDM + disulfiram group (n = 8). ^*^*P* < 0.05; ***P* < 0.01; ****P* < 0.001; *****P* < 0.0001, Wilcoxon rank-sum test.
